# Human TRUB1 is a highly conserved pseudouridine synthase responsible for the formation of Ψ55 in mitochondrial tRNA^Asn^, tRNA^Gln^, tRNA^Glu^ and tRNA^Pro^

**DOI:** 10.1093/nar/gkac698

**Published:** 2022-08-26

**Authors:** Zidong Jia, Feilong Meng, Hui Chen, Gao Zhu, Xincheng Li, Yunfan He, Liyao Zhang, Xiao He, Huisen Zhan, Mengquan Chen, Yanchun Ji, Meng Wang, Min-Xin Guan

**Affiliations:** Division of Medical Genetics and Genomics, The Children's Hospital, Zhejiang University School of Medicine and National Clinical Research Center for Child Health, Hangzhou, Zhejiang, China; Institute of Genetics, Zhejiang University School of Medicine, Hangzhou, Zhejiang, China; Division of Medical Genetics and Genomics, The Children's Hospital, Zhejiang University School of Medicine and National Clinical Research Center for Child Health, Hangzhou, Zhejiang, China; Institute of Genetics, Zhejiang University School of Medicine, Hangzhou, Zhejiang, China; Institute of Genetics, Zhejiang University School of Medicine, Hangzhou, Zhejiang, China; Institute of Genetics, Zhejiang University School of Medicine, Hangzhou, Zhejiang, China; Institute of Genetics, Zhejiang University School of Medicine, Hangzhou, Zhejiang, China; Institute of Genetics, Zhejiang University School of Medicine, Hangzhou, Zhejiang, China; Institute of Genetics, Zhejiang University School of Medicine, Hangzhou, Zhejiang, China; Institute of Genetics, Zhejiang University School of Medicine, Hangzhou, Zhejiang, China; Institute of Genetics, Zhejiang University School of Medicine, Hangzhou, Zhejiang, China; Department of Lab Medicine, Wenzhou Hospital of Traditional Chinese Medicine, Wenzhou, Zhejiang, China; Division of Medical Genetics and Genomics, The Children's Hospital, Zhejiang University School of Medicine and National Clinical Research Center for Child Health, Hangzhou, Zhejiang, China; Institute of Genetics, Zhejiang University School of Medicine, Hangzhou, Zhejiang, China; Division of Medical Genetics and Genomics, The Children's Hospital, Zhejiang University School of Medicine and National Clinical Research Center for Child Health, Hangzhou, Zhejiang, China; Institute of Genetics, Zhejiang University School of Medicine, Hangzhou, Zhejiang, China; Division of Medical Genetics and Genomics, The Children's Hospital, Zhejiang University School of Medicine and National Clinical Research Center for Child Health, Hangzhou, Zhejiang, China; Institute of Genetics, Zhejiang University School of Medicine, Hangzhou, Zhejiang, China; Zhejiang Provincial Key Laboratory of Genetic and Developmental Disorders, Hangzhou, Zhejiang, China; Joint Institute of Genetics and Genome Medicine between Zhejiang University and University of Toronto, Hangzhou, Zhejiang, China

## Abstract

Pseudouridine (Ψ) at position 55 in tRNAs plays an important role in their structure and function. This modification is catalyzed by TruB/Pus4/Cbf5 family of pseudouridine synthases in bacteria and yeast. However, the mechanism of TRUB family underlying the formation of Ψ55 in the mammalian tRNAs is largely unknown. In this report, the CMC/reverse transcription assays demonstrated the presence of Ψ55 in the human mitochondrial tRNA^Asn^, tRNA^Gln^, tRNA^Glu^, tRNA^Pro^, tRNA^Met^, tRNA^Leu(UUR)^ and tRNA^Ser(UCN)^. *TRUB1* knockout (KO) cell lines generated by CRISPR/Cas9 technology exhibited the loss of Ψ55 modification in mitochondrial tRNA^Asn^, tRNA^Gln^, tRNA^Glu^ and tRNA^Pro^ but did not affect other 18 mitochondrial tRNAs. An *in vitro* assay revealed that recombinant TRUB1 protein can catalyze the efficient formation of Ψ55 in tRNA^Asn^ and tRNA^Gln^, but not in tRNA^Met^ and tRNA^Arg^. Notably, the overexpression of *TRUB1* cDNA reversed the deficient Ψ55 modifications in these tRNAs in *TRUB1^KO^* HeLa cells. TRUB1 deficiency affected the base-pairing (18A/G-Ψ55), conformation and stability but not aminoacylation capacity of these tRNAs. Furthermore, TRUB1 deficiency impacted mitochondrial translation and biogenesis of oxidative phosphorylation system. Our findings demonstrated that human TRUB1 is a highly conserved mitochondrial pseudouridine synthase responsible for the Ψ55 modification in the mitochondrial tRNA^Asn^, tRNA^Gln^, tRNA^Glu^ and tRNA^Pro^.

## INTRODUCTION

Nucleotide modifications of transfer RNA (tRNA) affect all aspects of tRNA structure and function (1–3). In mammalian mitochondria, 18 types of nucleotide modifications occur in the 137 positions of 22 tRNA species, encoded by mitochondrial DNA (mtDNA) (4–6). The nucleotides at positions 34 and 37 at anticodon loop of tRNAs are more prone to be modified than those at other positions of tRNAs and impact the stabilization of anticodon structure, fidelity and efficiency of translation (7–12). The nucleotide modifications of tRNAs were catalyzed by a series of tRNA modifying enzymes, encoded by nuclear genome, synthesized in cytosol and subsequently imported into mitochondria (13–14). The biosynthesis of τm^5^s^2^U34 modification of tRNA^Lys^, tRNA^Glu^ and tRNA^Gln^ was catalyzed by tRNA modifying enzymes GTPBP3, MTO1 and TRMU (15–18). The m^1^G37 modification in the tRNA^Ala^, tRNA^Leu(CUN)^, tRNA^Pro^ and tRNA^Gln^ is synthesized by tRNA methyltransferase 5 (TRMT5), while the biosynthesis of i^6^A37 in the tRNA^Cys^, tRNA^Ser(UCN)^, tRNA^Phe^, tRNA^Trp^ and tRNA^Tyr^ was catalyzed by tRNA dimethylallyltransferase (TRIT1) (4,^19^,^20^).

Of core modifications, the pseudouridine (Ψ) at position 55 of TΨC arm plays an important role in the structure and function of tRNAs including proper folding and stability, and translation (21–23). In particular, the Ψ55 forms a tertiary base pair with the 18A/G in the D-loop and stabilizes the L-shaped tRNA structure (21,22). The defects in pseudouridinylation at position 55 at the TΨC loop of human mitochondrial tRNA^Glu^ caused by deafness and diabetes-associated m.14692A > G mutation resulted in the destabilization of base pairing (18A-Ψ55), improper folding, and instability of tRNA^Glu^ (23). In bacteria and yeast, the biosynthesis of pseudouridine at position U55 of tRNAs is catalyzed by TruB/Pus4/Cbf5 family of pseudouridine synthases (24–27). In human, TRUB family included at least three members: TRUB1, TRUB2 and Cbf5/DKC1 sharing highly conserved active site consensus sequences HXGXLD (27–30). Cbf5/DKC1 functions in the ribosomal biogenesis through ribosomal RNA (rRNA) pseudouridylation, splicing, and telomere maintenance (29). TRUB2 is involved in mitochondrial mRNA pseudouridylation, regulates 16S rRNA and mitochondrial translation (30–32). TRUB1 has tRNA Ψ55 synthase activity and is primarily present in the nucleus (32,33). However, the roles of TRUB1 in the formation of Ψ55 in the mitochondrial tRNAs are unknown. In this study, the mapping pseudouridines in 22 mitochondrial tRNAs and 4 cytoplasmic tRNAs by carbodiimide (CMC) modification/reverse transcription approach revealed the presence of Ψ55 in tRNA^Asn^, tRNA^Gln^, tRNA^Glu^, tRNA^Pro^, tRNA^Met^, tRNA^Leu(UUR)^ and tRNA^Ser(UCN)^ (34). To gain the roles of TRUB1 in biosynthesis of the Ψ55 in the tRNAs, we used CRISPR/Cas9 genomic editing approach in HeLa cells to produce the targeted deletions in *TRUB1* gene. The *TRUB1* knock-out (KO) (*TRUB1*^KO^) cell lines exhibited the complete loss of Ψ55 modification in mitochondrial tRNA^Asn^, tRNA^Gln^, tRNA^Glu^ and tRNA^Pro^. An *in vitro* enzymatic activity of TRUB1 was assayed with unmodified tRNA^Asn^, tRNA^Gln,^ tRNA^Met^ and tRNA^Arg^ generated by *in vitro* transcription as substrates using recombinant TRUB1. To verify the defects of *TRUB1* knock-out in the HeLa cells, we transferred a plasmid carrying the full-length *TRUB1* cDNA into the *TRUB1*^KO^ cell lines. These *TRUB1*^KO^ cell lines were then evaluated for the effects of TRUB1 deficiency on the conformation, stability, and aminoacylation capacity of mitochondrial tRNAs. These cell lines were then assessed for the effects of the TRUB1 deficiency on mitochondrial translation and the biogenesis of oxidative phosphorylation (OXPHOS) system.

## MATERIALS AND METHODS

### Construction of *TRUB1*^KO^ cell lines

The HeLa cells were grown in Dulbecco's Modified Eagle Medium (DMEM) (Life Technologies) (containing 4.5 mg of glucose, 0.11 mg pyruvate/ml and 50 μg of uridine/ml), supplemented with 5% FBS. *TRUB1*^KO^ Hela cell lines were constructed using the CRISPR-Cas9 system 458 plasmid (Addgene) containing the sgRNAs (35–37). The sgRNAs were designed using the CRISPR design tool (http://crispr.mit.edu) to minimize potential off-target effects. The sequences of sgRNAs that ultimately produced successful deletion clones were 5′-CACCGCACGGCGAACACGCCGCTCA-3′ and 5′-AAACTGAGCGGCGTGTTCGCCGTGC-3′. HeLa cells were transfected with Lenti CRISPR 458 plasmid (Addgene) containing the sgRNAs using jetPRIME (Polyplus-transfection SA, Illkirch, France), according to the manufacturer's instructions. After 24 hours, the cell cultures in the same media were treated with 1μg/ml of puromycin for three days. The cells were then collected and plated in the DMEM, supplemented with 10% FBS. Subsequently, cells were cloned by limiting dilution and individual clones were isolated.

The genotyping for the *TRUB1* mutations in each clone was performed by PCR amplification of 400 bp fragment spanning partial promoter region and exon 1 (RefSeq NC_000010.11) and followed by Sanger sequence analysis. The forward and reverse primers for this genotyping analysis were 5′-GCTTCTGAGGCGGCGGTGGTGTCTTC-3′ and 5′-AATGGTCAGTCTCCCTTTCCCCTCCTTTTG-3′, respectively.

For the rescuing of *TRUB1^KO^* cells, the full-length coding region of *TRUB1* cDNA was obtained by RT-PCR amplification using the high fidelity Pfu DNA polymerase (Promega) and total RNA isolated from HeLa cells as template, with primers with *Nhe*I site: 5′-TTGCTAGCCGCCACCATGGCCGCTTCTGAGGCGG-3′ (nt.60–78) and *EcoR*I site: 5′-TTGAATTCTTGTCATTCTGCATCTGCACACAGGA-3′(nt.1152–1177) (GenBank accession no. NM_139169.5). The PCR products were cloned by using the TA Cloning Kit (TaKaRa), analyzed by Sanger sequencing and then subcloned into a pCDH-puro-cMyc Vector (Addgene plasmid 46970). The pCDH-puro-cMyc Vector containing *TRUB1* cDNA were co-transfected with the packaging vectors pMD2.G (Addgene) and psPAX2 (Addgene) into 293T cell line by Lipofectamine 3000 (Invitrogen) to produce virus. Two days following transfection, viral supernatants were collected and used to infect the *TRUB1*^KO^ cells (38).

### Mitochondrial location analysis

Immunofluorescence experiments were performed as detailed previously (36,37). HeLa cells were cultured on cover glass slips (Thermo Fisher), fixed in 4% formaldehyde for 15 min, permeabilized with 0.2% Triton X-100, blocked with 5% Fetal Bovine Serum (FBS) for 1h, and immunestained with an anti-TOM20 antibody (Abcam) and anti-FLAG antibody (Abcam) overnight at 4°C. The cells were then incubated with Alex Fluor 594 goat anti-mouse IgG (H&L) and Alex Fluor 488 goat anti-rabbit IgG (H&L) (Thermo Fisher), stained with 4′,6-daimidino-2-phenylindole (DAPI) (Invitrogen) for 15 minutes, and mounted with Fluoromount (Sigma-Aldrich). Cells were examined using a confocal fluorescence microscope (Olympus Fluoview FV1000, Japan) with three lasers (Ex/Em = 550/570, 492/520 and 358/461 nm).

### Detection of pseudouridine residues in tRNAs using CMC modification/reverse transcription assay

RNAs for CMC treatment were total enriched small RNA including mitochondrial and cytosolic tRNAs, isolated from various cell lines by using RNAiso for Small RNA kit (TaKaRa). Twenty micrograms of RNAs were incubated with 160 mM 1-cyclohexyl-(2-morpholinoethyl) carbodiimide metho-p-toluene sulfonate (CMCT) for 20 min at 37°C to allow for carbodiimide (CMC) modification of Ψ residues (34). The reaction mixtures contain 7 M urea, 4 mM EDTA, 50 mM Bicine, pH 8.5. The modified RNAs were then precipitated by adding 2 μl of Pellet Paint Co-Precipitant, 50 μl of 3 M sodium acetate, pH 5.5, and triple volume of ethanol, incubated at least 2 h at –20°C before centrifuging at 12 000 rpm for 30 min. The RNA pellets were dissolved in 1 M sodium carbonate, pH 10.4, incubated for 4 hours at 37°C, and precipitated again as described above. Primescript II 1st Strand cDNA Synthesis Kit (TaKaRa) was used for reverse transcription with digoxigenin (DIG)-labeled oligodeoxynucleotide probes specific for 22 mitochondrial tRNAs and 4 cytosolic tRNAs (Supplemental Table S1). RNase A was added to the extension reaction to remove the mitochondrial RNA. The DNA was then precipitated with ethanol at –20°C overnight after phenol extraction. Two micrograms of DNA samples were applied onto 15% polyacrylamide, 7 M urea electrophoresis gel and electroblotted onto a positively charged nylon membrane.

### Western blot analysis

Western blot analysis was performed as detailed elsewhere (39). Twenty micrograms of total proteins obtained from various cell lines were electrophoresed through 10% bis–Tris SDS-polyacrylamide gels. Afterward, the gels were electroblotted onto polyvinylidene difluoride (PVDF) membrane for hybridization. The antibodies used for this investigation were from Invitrogen [TRUB1 (PA5-36003) and ND4L(PA5-68242)], Abcam [ND1(ab74257), ND3(ab170681), ND5(ab92624), TOMM20/TOM20(ab56783), SDHB(ab14714), UQCRC2(ab14745) and TUBULIN (ab6046)], Novus [ND4(NBP2-47365)], ABclonal [NDUFA1(A20940) and NDUFA10(A10123)] and Proteintech [FLAG (80010-1-RR), NDUFS1(12444-1-AP), UQCRFS1(18443-1-AP), COXIV(66110-1-Ig), COX17 (11464-1-AP), CYTB (55090-1AP), CO2 (55070-1-AP), ATP8 (26723-1-AP), ATP5B (17247-1-AP), ATP5F1(15999-1-AP) and GAPDH (60004-1-Ig)]. Peroxidase Affinipure goat anti-mouse IgG and goat anti-rabbit IgG (Beyotime) were used as secondary antibodies, and protein signals were detected using the ECL system (CWBIO). The quantification of density in each band was performed as detailed previously (38).

### *In vitro* transcription of mitochondrial tRNAs

tRNA transcripts as substrates for enzymatic reactions were produced as described previously (23,40). The mitochondrial tRNA^Asn^, tRNA^Gln^, tRNA^Met^ and tRNA^Arg^ were generated by *in vitro* transcription by T7 RNA polymerase (Promega) using synthetic DNA oligonucleotides as templates. Forward oligos for all tRNAs contained T7 RNA polymerase promoter sequences. The transcripts were purified by 10% polyacrylamide gel electrophoresis. The sequences of oligonucleotides were 5′-GCTAATACGACTCACTATATAGATTGAAGCCAGTTGATTAGGGTGCTTAGCTGTTAACTAAGTGTTTGTGGGTTTAAGTCCCATTGGTCTAGCCA-3′ and 5′-TGGCTAGACCAATGGGACTTAAACCCACAAACACTTAGTTAACAGCTAAGCACCCTAATCAACTGGCTTCAATCTATATAGTGAGTCGTATTAGC-3′ for tRNA^Asn^, 5′-GCTAATACGACTCACTATATAGGATGGGGTGTGATAGGTGGCACGGAGAATTTTGGATTCTCAGGGATGGGTTCGATTCTCATAGTCCTAGCCA-3′ and 5′-TGGCTAGGACTATGAGAATCGAACCCATCCCTGAGAATCCAAAATTCTCCGTGCCACCTATCACACCCCATCCTATATAGTGAGTCGTATTAGC-3′ for tRNA^Gln^, 5′-GCTAATACGACTCACTATAAGTAAGGTCAGCTAAATAAGCTATCGGGCCCATACCCCGAAAATGTTGGTTATACCCTTCCCGTACTACCA -3′ and 5′-TGGTAGTACGGGAAGGGTATAACCAACATTTTCGGGGTATGGGCCCGATAGCTTATTTAGCTGACCTTACTTATAGTGAGTCGTATTAGC -3′ for tRNA^Met^, 5′-GCTAATACGACTCACTATATGGTATATAGTTTAAACAAAACGAATGATTTCGACTCATTAAATTATGATAATCATATTTACCAACCA -3′ and 5′-TGGTTGGTAAATATGATTATCATAATTTAATGAGTCGAAATCATTCGTTTTGTTTAAACTATATACCATATAGTGAGTCGTATTAGC -3′ for tRNA^Arg^.

### Expression and purification of recombinant TRUB1 protein

The open reading frames of *TRUB1* gene (GenBank accession number: NM_139169) were amplified through RT-PCR by using primers (5′-CGGAATTCATGGCCGCTTCTGAGG-3′ and 5′-GCGTCGACTCAGTGGTGGTGGTGG-3′) containing an *EcoR*I restriction site added to the 5′ end and a *Sal*I restriction site added to the 3′ end. The PCR products were digested and ligated into a pMAL-c2E-derived vector (pMAL-c2E-TEV-His) between the *Eco*RI and *Sal*I sites, which produces a maltose binding protein (MBP)-fused protein containing a TEV cleavage site between MBP and fused partner and a His-tag fused at the C terminus of recombinant protein (41). The pMAL-c2E-TRUB1 was transformed into *Escherichia coli* DH5α and then incubated at 37°C for 4h in Luria-Bertani medium containing ampicillin (100 μg/ml) after the addition of 1 mM isopropyl β-d-1-thiogalactopyranoside (IPTG). The recombinant TRUB1 protein was separated via SDS-PAGE stained with Coomassie Brilliant Blue and purified through High Affinity Ni-Charged Resin FF in accordance with the manufacturer's manual (Genscript). The TEV Protease (Beyotime) was used to digest the fusion protein TRUB1-MBP to release the MBP tag.

### *In vitro* assays for pseudouridylation activity of recombinant human TRUB1

The pseudouridylation activity of purified recombinant TRUB1 was assayed at 37°C for 1 h in a reaction mixture (50 mM HEPES, pH 7.5, 100 mM NH_4_Cl, 5 mM Mg(OAc)_2_, 5 mM DTT, 400 units/mL RNasin) with 500 nM of *in vitro* transcribed tRNAs as substrates (24). The reaction was terminated by phenol-chloroform extraction and RNAs were recovered by ethanol precipitation. The modified tRNAs was then treated and analyzed for the Ψ modification as described as above.

### Mitochondrial tRNA analysis

Total cellular RNAs were obtained by using TOTALLY RNA^TM^ kit (Ambion) from intact cells from various cell lines, as detailed elsewhere (42). For tRNA Northern blot analysis, 5 μg of total cellular RNAs were electrophoresed through a 10% polyacrylamide gel without (native gel) or with (denature gel) 8M urea in Tris-borate-EDTA buffer (TBE) (after heating the sample at 65°C for 10 min), and then electroblotted onto a positively charged nylon membrane for the hybridization analysis with DIG-labeled oligodeoxynucleotide probes for tRNA^Asn^, tRNA^Gln^, tRNA^Glu^, tRNA^Pro^, tRNA^Ser(UCN)^, tRNA^Leu(UUR)^, tRNA^Ser(AGY)^, tRNA^Leu(CUN)^, tRNA^Met^ and 5S rRNA were as detailed previously (43–46). DIG-labeled oligodeoxynucleotides were generated by using DIG oligonucleotide tailing kit (Roche). The hybridization and quantification of density in each band were performed as detailed elsewhere (44–46).

For the aminoacylation assays, mitochondrial RNAs were obtained from mitochondria isolated from various cell lines (∼2.0 × 10^7^ cells), under acid conditions as detailed elsewhere (43,47). Five micrograms of mitochondrial RNAs were electrophoresed at 4°C through an acid (pH 5.0) 10% polycrylamide/8 M urea gel to separate the charged and uncharged tRNAs detailed elsewhere (46,47). To further distinguish nonaminoacylated tRNA from aminoacylated tRNA, samples of tRNAs were deacylated by being heated for 10 min at 60°C (pH 8.3) and then run in parallel. The gels were then electroblotted onto a positively charged nylon membrane (Roche) for the hybridization analysis with oligodeoxynucleotide probes as described above. Quantification of density in each band was performed as detailed previously (46,47).

The S1 nuclease cleavage analysis was performed as detailed elsewhere (18,46,48). In brief, 2 μg of total RNAs were incubated with 1 μg/μl total yeast tRNA and 1U/μl S1 nuclease (Thermofisher) in the 5 μl reaction buffer containing 40 mM sodium acetate (pH 4.5), 300 mM NaCl and 2 mM ZnSO_4_. Reaction mixtures were incubated at 28°C for indicated times and quenched by adding 5μl loading buffer. Samples were electrophoresed through a 10% denaturing polyacrylamide gel with 8 M urea and then electroblotted onto a positively charged nylon membrane for hybridization analysis with DIG-labeled oligodeoxynucleotide probes as described above.

### Blue native polyacrylamide gel electrophoresis (BN-PAGE) and in-gel activity assays

BN-PAGE was performed on mitochondrial protein extracted from various cell lines as detailed elsewhere (49,50). For in-gel activity assays, samples containing 30 μg of total mitochondrial proteins were separated on 3 to 12% Bis–Tris Native PAGE gel. The native gels were prewashed in cold water and then incubated with the substrates of complex I, complex II, complex IV and complex V at room temperature as described elsewhere (49,50). After stopping reaction with 10% acetic acid, gels were washed with water and scanned to visualize the activities of respiratory chain complexes.

### Statistical analysis

Statistical analysis was carried out using the unpaired, two-tailed Student's t-test contained in the Microsoft-Excel program or Macintosh (version 2007). Differences were considered significant at a *P* < 0.05.

## RESULTS

### The presence of Ψ55 in mitochondrial tRNA^Asn^, tRNA^Gln^, tRNA^Glu^, tRNA^Met^, tRNA^Leu(UUR)^, tRNA^Ser(UCN)^ and tRNA^Pro^

**Figure 1.**
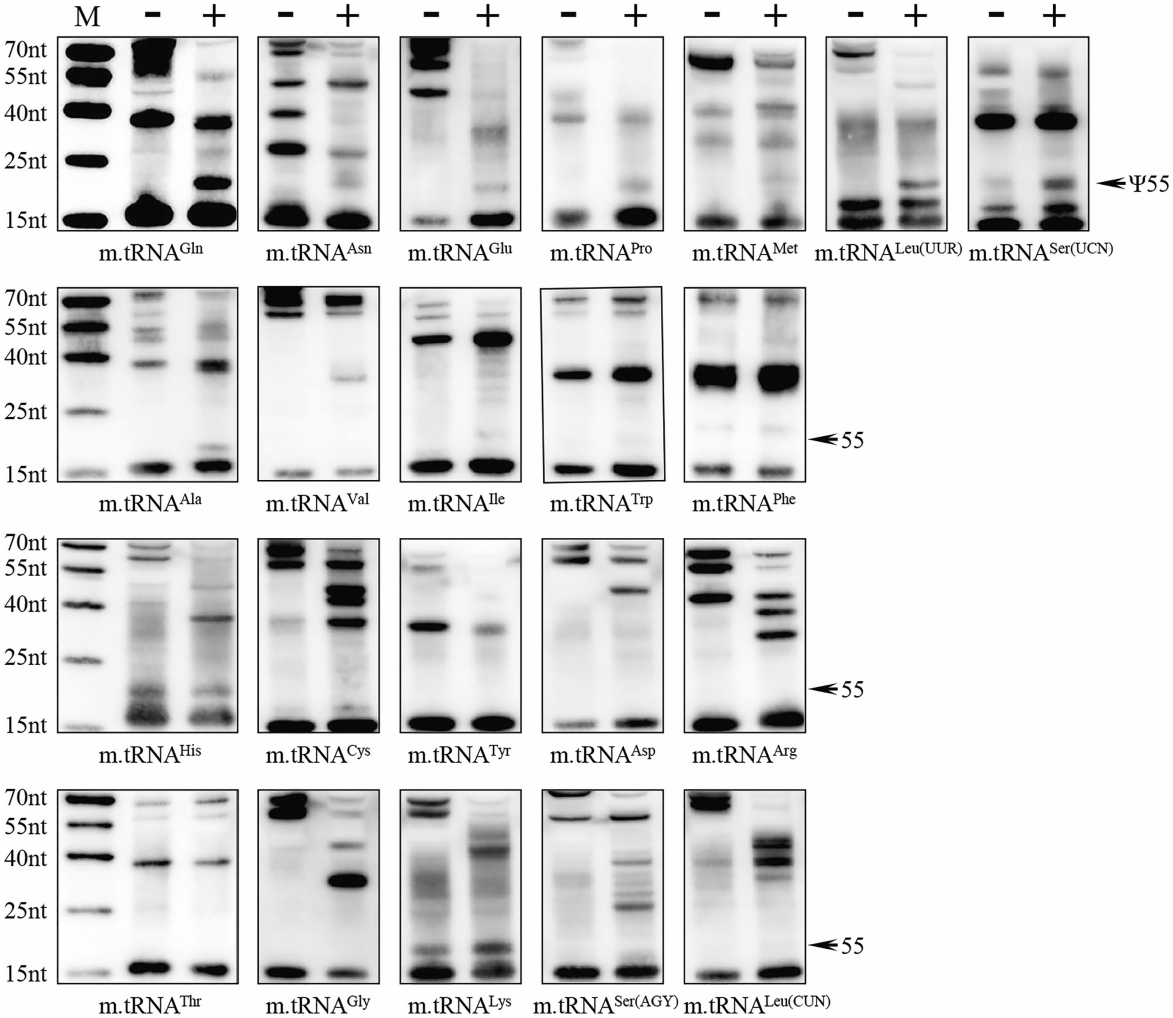
Pseudouridine sequencing of mitochondrial tRNAs. Primer extension analysis of all the 22 mitochondrial tRNAs after CMCT treatment of small rRNA enriched total RNAs. Total RNAs isolated from HeLa cells were treated with (+) or without (−) CMCT, followed by alkali (OH^−^) treatment. Reverse transcription was carried out using DIG- labeled primers to identify the stops caused by CMC-pseudouridine. The arrow indicates a strong stop at Ψ55. M: marker, DIG-labeled oligonucleotides of variable length.

To examine the presence of Ψ55 in human mitochondrial tRNAs, we subjected total enriched RNAs from HeLa cells to the CMC/reverse transcription assays with DIG-labeled oligonucleotide probes specific for 22 mitochondrial tRNAs including tRNA^Asn^, tRNA^Gln^, tRNA^Glu^, tRNA^Pro^, tRNA^Met^, tRNA^Leu(UUR)^ and tRNA^Ser(UCN)^. This approach involved CMC adduct formation with U, G and pseudouridine followed by mild alkali to remove the adduct from U and G, but not from the *N*3-[*N*-cyclohexyl-*N*'-β-(4-methyl-morpholinium) ethylcarbodiimide]-Ψ (N_3_-CMC-Ψ) (34). This yielded the attenuation of primer reverse transcription, causing a stop band one residue 3′ to the pseudouridine on sequence gel. As shown in Figure 1, the Ψ55 modification was detected in the mitochondrial tRNA^Asn^, tRNA^Gln^, tRNA^Glu^, tRNA^Pro^, tRNA^Met^, tRNA^Leu(UUR)^ and tRNA^Ser(UCN)^ but not in other 15 mitochondrial tRNAs.

### Human TRUB1 is a conserved pseudouridine synthase localized in mitochondrion

Human *TRUB1* encodes a 349 amino acid protein with a putative mitochondrial target sequence at the N-terminus, predicted by Mitoprot program (51). TRUB1 contains the VFAVHKPKGPTSA box in positions 71–83 corresponding to motif I that is involved in conserving protein structure and GGTLDS AARGVLVV in positions 117–130, including the highly conserved 121D residue, characterized motif II (involved in uridine recognition and in catalytic function) (28). Alignment of TRUB1 with its homologs of other organisms, including *Mus musculus, Rattus norvegicus, Sus scrofa, Canis lupus familiaris, Gallus gallus, Xenopus tropicalis, Danio rerio* and*Escherichia coli* displayed an extensive conservation of protein sequence. In particular, TRUB1 shares an overall amino acid identity of 79.4%, 81.4%, 86.7%, 92.3%, 58.9%, 51.0%, 52.7% and 20.1% with *M. musculus, R. norvegicus, S.scrofa, C. familiaris, G. gallus, X. tropicalis, D. rerio* and *E. coli*, respectively (Supplementary Figure S1).

**Figure 2.**
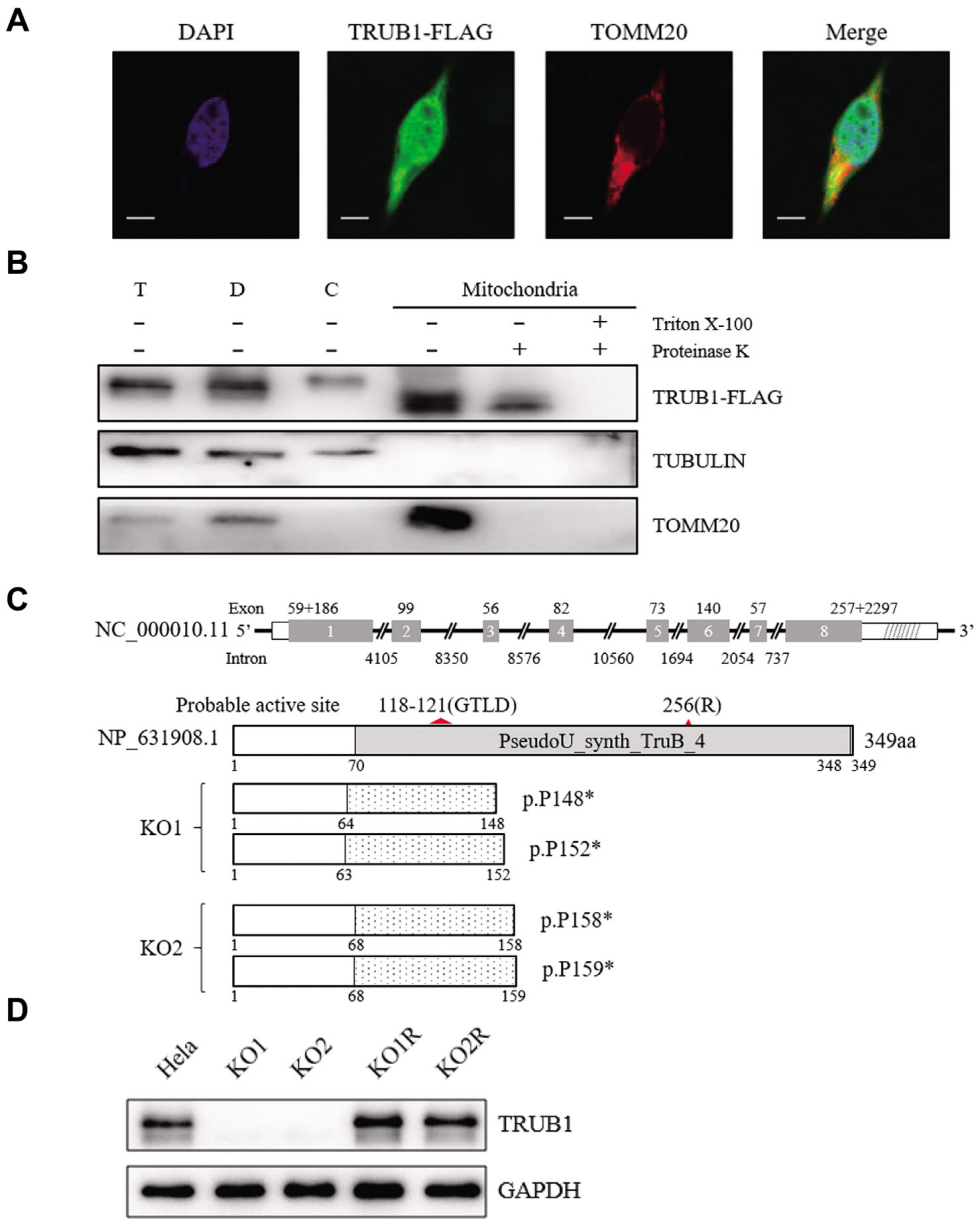
Subcellular location and generation of TRUB1 knockout HeLa cell lines using CRISPR/Cas9 system. (**A**) Subcellular localization of TRUB1 by immunofluorescence in HeLa cells. TRUB1-FLAG (shown in green), TOMM20 (shown in red), and DAPI (shown in blue). Scale bar: 10 μm. (**B**) Subcellular localization of TRUB1 by Western blot with anti-FLAG, TOMM20 (mitochondrial) and TUBULIN (cytosol). T, total cell lysate; D, debris; C, cytosol; Mito, mitochondria. Isolated mitochondria were treated with (+) or without (–) 1% Triton X-100 followed by proteinase K digestion, respectively. (**C**) Schematic representation of TRUB1 and its truncated proteins. Shaded boxes indicate the PseudoU_synth_TruB_4 domain of TRUB1. Red triangle shows the probable active site. Deletion or insertion resulting in truncated proteins. (**D**) Western blot analysis. Twenty micrograms of total cellular proteins of each cell line were electrophoresed through and hybridized with antibodies specific for TRUB1 or with GAPDH as a loading control. KO1 and KO2 represented two *TRUB1* knockout - cell lines; KO1R and KO2R represented KO1 and KO2 expressing wild type *TRUB1* cDNA.

To examine the subcellular localization of human TRUB1, we transfected a *TRUB1* construct with the FLAG-tag at the C-terminus of this protein into Hela cells, first examined subcellular localization by immunofluorescence analysis. Using antibodies against FLAG and TOMM20, a mitochondrial protein, we observed overlaps of both fluorescence signals in the transfected cells (Figure 2A). To further determine the mitochondrial localization of TRUB1, a FLAG-tagged version of TRUB1was transiently expressed with in the HeLa cells. Cellular fraction experiment of HeLa cells revealed that the exogenous TRUB1 was enriched within mitochondrial fractions, along with outer mitochondrial membrane protein TOMM20, and present in cytosol, along with the cytosolic protein tubulin (Figure 2B). These indicated that TRUB1 localizes at mitochondria but not exclusively. These suggested that TRUB1 plays a role in the formation of Ψ55 in tRNAs not only at nucleus but also mitochondrion.

### Generation of *TRUB1* knockout HeLa cell lines

To gain overall information on how the *TRUB1* deficiency affected the synthesis of Ψ55 in mitochondrial tRNAs, we used CRISPR/Cas9 genomic editing approach to produce the targeted deletion in *TRUB1* gene in the HeLa cell line. This led to the generation of two *TRUB1* knockout (KO) cell lines carrying composite deletions (Figure 2C, Supplemental Figure S2). The *TRUB1*^KO^^1^ cell line harbored two alleles with a 32 bp or 20 bp deletion in the exon 1 of *TRUB1*, respectively. The 32 bp deletion resulted in a frameshift from codon 64, the introduction of a premature stop at codon 148 (p.Pro148*), and truncated protein with 147 resides, while the 20 bp deletion yielded a frameshift from codon 63, the introduction of a premature stop at codon 152 (p.Pro152*), and truncated protein with 151 amino acids. The *TRUB1*^KO2^ cell line carried two alleles, which were generated by introducing a 2 bp deletion or a 1 bp insertion in the exon 1 of *TRUB1*, respectively. The 2 bp deletion yielded a frameshift from codon 58, the introduction of a premature stop at codon 158 (p.Pro158*), and truncated protein with 157 amino acids, while the 1 bp deletion resulted in a frameshift from codon 68, the introduction of a premature stop at codon 159 (p.Pro159*), and truncated protein with 158 amino acids. These alleles were confirmed by Sanger sequencing (Supplemental Figure 2), and Western blot analysis (Figure 2D). To further confirm the ablation of TRUB1 in HeLa cells, we transferred a plasmid carrying the full-length *TRUB1* cDNA into two *TRUB1*^KO^ cell lines. Indeed, the overexpression of *TRUB1* cDNA reversed the levels of TRUB1 in the *TRUB1*^KO^ cell lines.

### TRUB1 deficiency caused the complete loss of Ψ55 in mitochondrial tRNA^Asn^, tRNA^Gln^, tRNA^Glu^ and tRNA^Pro^

**Figure 3.**
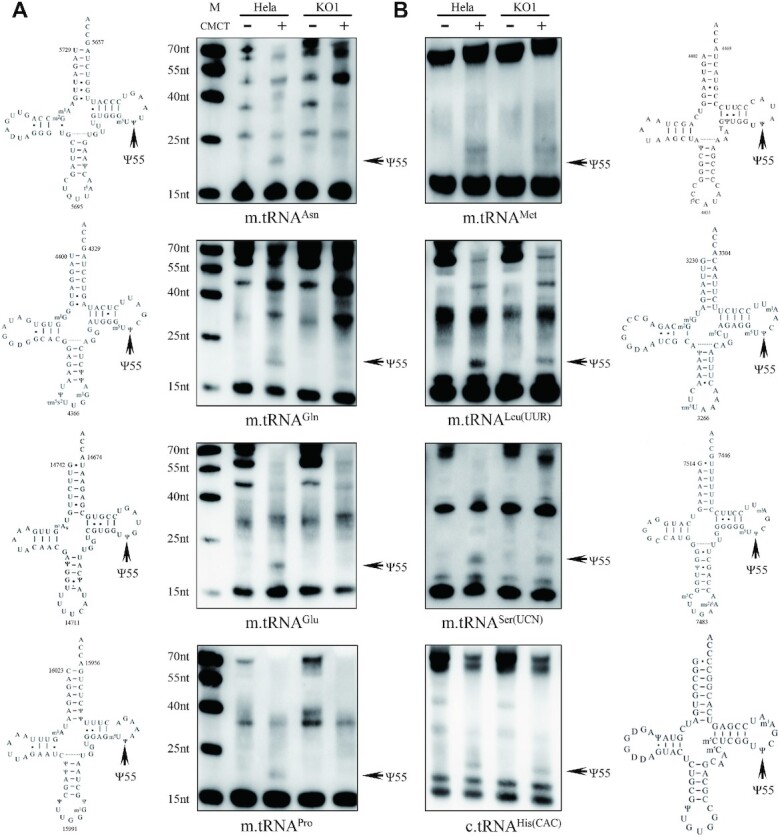
Pseudouridine sequencing of mitochondrial tRNAs. The cloverleaf structures derived from Suzuki *et al.* (4) and CMCT-primer extension analysis of enriched small RNAs in TRUB1 knockout (KO1) and wild type cell lines. Twenty micrograms of enriched small RNAs isolated from WT and *TRUB1*^KO1^ cell lines were incubated with CMCT for CMC modification of Ψ residues (+) or without (—) CMCT, followed by alkali (OH^−^) treatment. Reverse transcription was carried out using DIG-labeled primers to identify the stops caused by CMC-pseudouridine. (**A**) TRUB1 deficiency caused the loss of Ψ55 in mitochondrial tRNA^Asn^, tRNA^Gln^, tRNA^Glu^ and tRNA^Pro^. (**B**) TRUB1 deficiency did not affect the Ψ55 in tRNA^Met^, tRNA^Leu(UUR)^, tRNA^Ser(UCN)^ and c.tRNA^His(CAC)^. The arrow indicates a strong stop at Ψ55. M: marker, DIG-labeled oligonucleotides of variable length.

To investigate whether TRUB1 catalyzes the synthesis of Ψ55 in mitochondrial tRNA^Asn^, tRNA^Gln^, tRNA^Glu^, tRNA^Pro^, tRNA^Met^, tRNA^Leu(UUR)^ and tRNA^Ser(UCN)^, we subjected total enriched small RNAs from *TRUB1*^KO^ and wild type (WT) cell lines to the CMC/reverse transcription with DIG-labeled oligonucleotide probes specific for mitochondrial tRNA^Asn^, tRNA^Gln^, tRNA^Glu^, tRNA^Pro^, tRNA^Met^, tRNA^Leu(UUR)^ and tRNA^Ser(UCN)^, as well as cytoplasmic tRNA^Thr(ACU)^, tRNA^Met(AUG)^, tRNA^Tyr(UAC)^ and tRNA^His(CAC)^, which contain Ψ55 modification (5). As shown in Figure 3A, the Ψ55 modification was not detected in mitochondrial tRNA^Asn^, tRNA^Gln^, tRNA^Glu^ and tRNA^Pro^ derived from the *TRUB1*^KO^ cell lines, but present in the WT cell line. However, the Ψ55 modification was still present in the mitochondrial tRNA^Met^, tRNA^Leu(UUR)^ and tRNA^Ser(UCN)^, as well as cytosolic tRNA^Thr(ACU)^, tRNA^Met(AUG)^, tRNA^Tyr(UAC)^ and tRNA^His(CAC)^ derived from both *TRUB1*^KO^ and WT cell lines (Figure 3B, supplemental Figure 3). These data demonstrated that TRUB1 is responsible for the formation of Ψ55 in the tRNA^Asn^, tRNA^Gln^, tRNA^Glu^ and tRNA^Pro^ but not in other mitochondrial tRNAs or 4 cytosolic tRNAs.

### An *in vitro* enzymatic activity assay of recombinant human TRUB1

**Figure 4.**
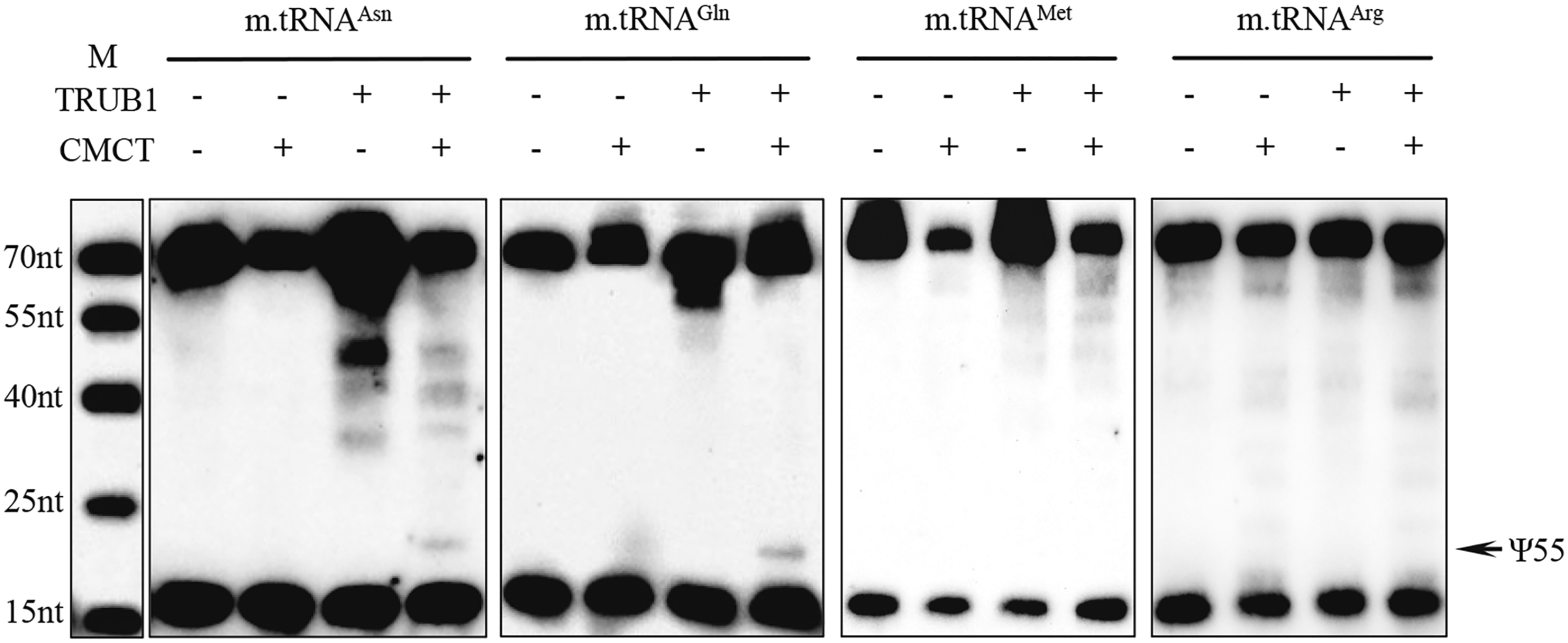
An *in vitro* enzymatic activity assay of recombinant human TRUB1. Unmodified mitochondrial tRNA^Asn^, tRNA^Gln^, tRNA^Met^ and tRNA^Arg^ synthesized by *in vitro* transcription were incubated with recombinant human TRUB1 and the reactions were stopped by phenol-chloroform extraction. The resultant modified tRNA samples were then subjected to primer extension using DIG-labeled primers. The strong stops in the reverse transcription of tRNA correspond to pseudouridine residues. Arrows indicated the Ψ at position 55 of tRNAs. M: marker, DIG-labeled oligonucleotides of variable length.

To further examine whether TRUB1 catalyzes the isomerization of U55 in mitochondrial tRNAs, we produced human recombinant TRUB1 protein using a prokaryotic expression system and mitochondrial tRNA^Asn^, tRNA^Gln^, tRNA^Met^ and tRNA^Arg^ as substrates by *in vitro* transcription. These unmodified tRNA^Asn^, tRNA^Gln^ (U55) transcripts together with tRNA^Met^ (U55) and tRNA^Arg^ (A55) transcripts as negative controls were incubated with recombinant TRUB1 protein to examine the formation of a single Ψ residue at position 55 in these tRNAs. As shown in Figure 4, the Ψ55 modification was detected in tRNA^Asn^ and tRNA^Gln^ transcripts (U55) in the presence of recombinant TRUB1 protein. However, the Ψ55 modification was not detected in the tRNA^Met^ (U55) and tRNA^Arg^ transcripts (A55) in the presence of recombinant TRUB1 protein. These results verified the essential role of TRUB1 in the formation of Ψ55 in tRNA^Asn^ and tRNA^Gln^, as well as tRNA^Glu^ and tRNA^Pro^.

### Abnormal conformation and instability of tRNA^Asn^, tRNA^Gln^, tRNA^Glu^ and tRNA^Pro^

**Figure 5.**
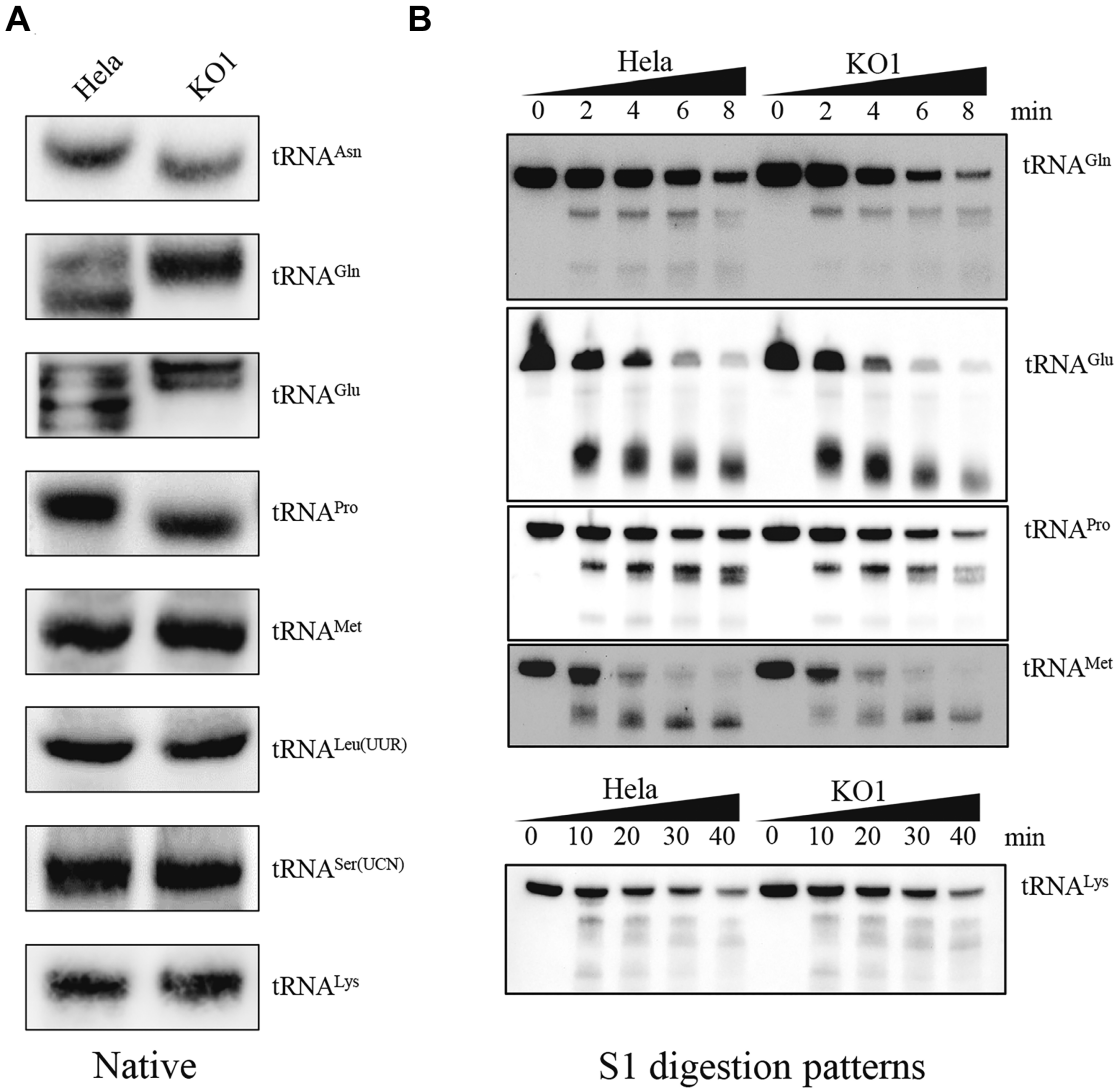
Analysis of mitochondrial tRNA conformation. (**A**) Northern blot analysis of tRNAs under native conditions. Two micrograms of total RNAs from *TRUB1*^KO1^ and wild type cell lines were electrophoresed through native polyacrylamide gel, electroblotted, and hybridized with DIG-labeled oligonucleotide probes for tRNA^Asn^, tRNA^Gln^, tRNA^Glu^, tRNA^Pro^, tRNA^Met^, tRNA^Leu(UUR)^, tRNA^Ser(UCN)^ and tRNA^Lys^, respectively. (**B**) S1 digestion patterns of tRNA^Gln^, tRNA^Glu^, tRNA^Pro^, tRNA^Met^ and tRNA^Lys^, purified from *TRUB1*^*KO1*^ and WT cell lines. Two micrograms of RNAs were used for the S1 cleavage reaction at various lengths (from 0 to 120 min). Cleavage products of tRNAs were resolved in 10% desaturating PAGE gels with 8 M urea, electroblotted and hybridized with 3′ end DIG-labeled oligonucleotide probes specific for tRNA^Gln^, tRNA^Glu^, tRNA^Pro^, tRNA^Met^ and tRNA^Lys^, respectively.

It was anticipated that the destabilization of base-pairing (18A/G-Ψ55) perturbed the structure and function of tRNA^Asn^, tRNA^Gln^, tRNA^Glu^ and tRNA^Pro^. To test if the *TRUB1* deficiency-induced loss of Ψ55 in tRNA^Asn^, tRNA^Gln^, tRNA^Glu^ and tRNA^Pro^ affects the conformation of these tRNAs, total RNAs from *TRUB1*^KO^ cell lines or those with overexpression of*TRUB1* cDNA and WT cell lines were electrophoresed through a 10% polyacrylamide gel (native condition) in Tris-borate-EDTA buffer and then electroblotted onto a positively charged nylon membrane for hybridization analysis with DIG-labeled oligodeoxynucleotide probes for tRNA^Asn^, tRNA^Gln^, tRNA^Glu^, tRNA^Pro^, tRNA^Met^, tRNA^Leu(UUR)^, tRNA^Ser(UCN)^ and tRNA^Lys^, respectively. As shown in Figure 5A, electrophoretic patterns showed that tRNA^Gln^, tRNA^Glu^ in *TRUB1*^KO^ cells migrated slower than those of wild type cells, while electrophoretic patterns in tRNA^Asn^, tRNA^Pro^ in *TRUB1*^KO^ cells migrated faster than those of wild type cells. In contrast, there were no difference of electrophoretic patterns in tRNA^Met^, tRNA^Leu(UUR)^, tRNA^Ser(UCN)^ and tRNA^Lys^ between *TRUB1*^KO^ and WT cell lines. These data suggested that the loss of Ψ55 in tRNA^Asn^, tRNA^Gln^, tRNA^Glu^ and tRNA^Pro^ changed the conformation of these tRNAs.

We further evaluated whether the loss of TRUB1 perturbed the structures of tRNAs by analyzing the sensitivity of tRNA^Gln^, tRNA^Glu^, tRNA^Pro^, tRNA^Met^ and tRNA^Lys^ from *TRUB1*^KO^ and WT cell lines to digestion with the nuclease S1. The resultantly digested-products from *TRUB1*^KO^ and WT cell lines were then followed by Northern blot analysis using tRNA probes that hybridized only to 3′ half tRNAs. As illustrated in Figure 5B, the tRNA^Gln^, tRNA^Glu^ and tRNA^Pro^ from *TRUB1*^KO^ cell lines were more sensitive to S1-mediated digestion than those from WT cell lines and exhibited remarkable differences in S1-mediated digestion patterns of tRNAs from WT cell lines. Conversely, there was no significant difference between the sensitivity of tRNA^Met^ and tRNA^Lys^ from *TRUB1*^KO^ and WT cell lines to digestion with the nuclease S1. These data validated that the inactivation of TRUB1 changed the conformation of mitochondrial tRNAs.

### No effect of steady-state levels and aminoacylation of mitochondrial tRNAs

**Figure 6.**
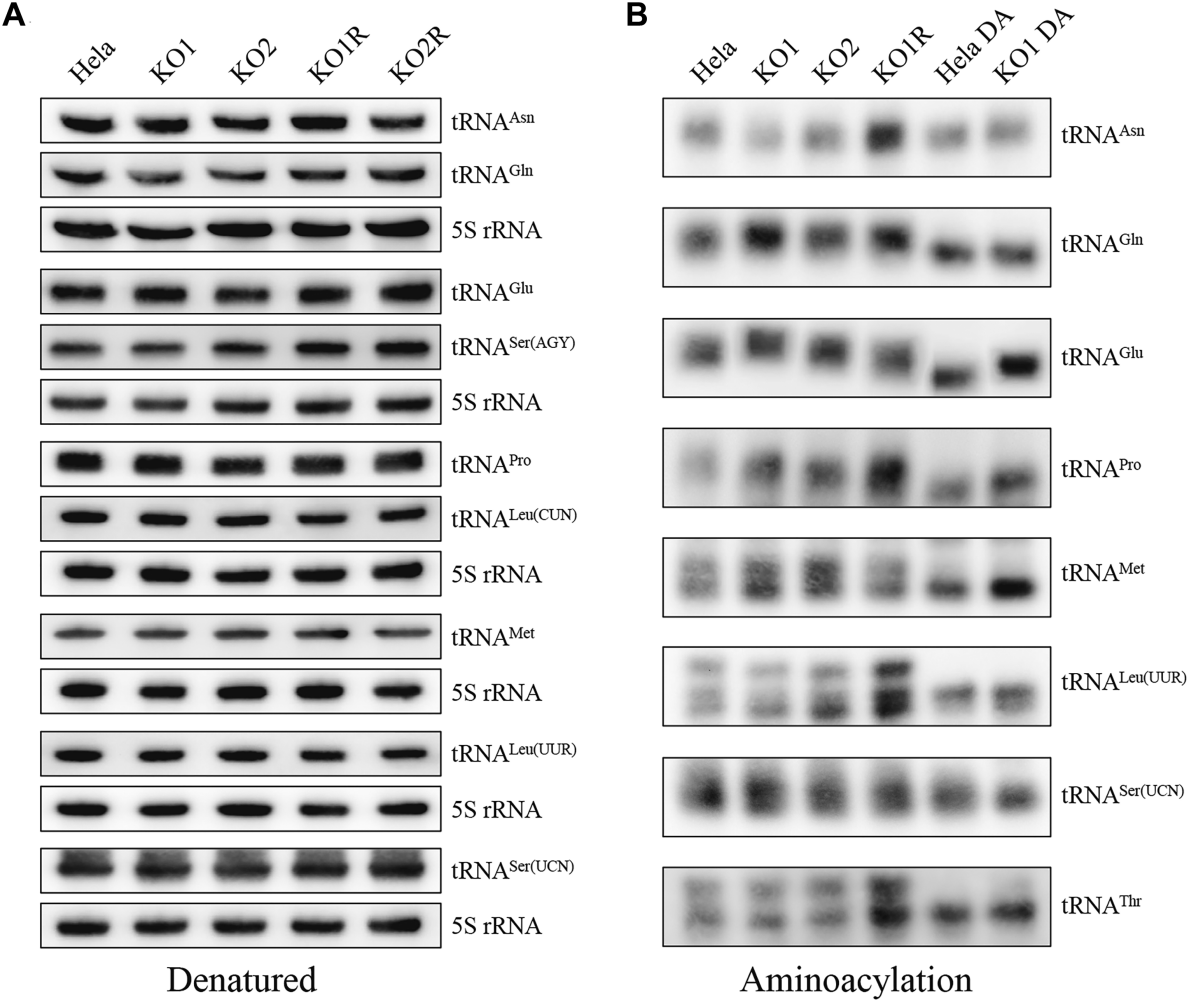
Analysis of steady state levels and aminoacylation of mitochondrial tRNA. (**A**) Northern blot analysis of tRNAs under denatured condition. Five micrograms of total cellular RNA from various cell lines were electrophoresed through a denaturing polyacrylamide gel, electroblotted, and hybridized with DIG-labeled oligonucleotide probes for tRNA^Asn^, tRNA^Gln^, tRNA^Glu^, tRNA^Ser(AGY)^, tRNA^Pro^, tRNA^Leu(CUN)^, tRNA^Met^, tRNA^Leu(UUR)^, tRNA^Ser(UCN)^ and 5S rRNA as a loading control, respectively. (**B**) Aminoacylation assays. Five micrograms of mitochondrial RNAs purified from various cell lines under acid conditions was electrophoresed at 4°C through an acid (pH 5.0) 10% polyacrylamide-8 M urea gel, electroblotted, and hybridized with a DIG-labeled oligonucleotide probe specific for the tRNA^Asn^, tRNA^Gln^, tRNA^Glu^, tRNA^Pro^, tRNA^Met^, tRNA^Thr^, tRNA^Leu(UUR)^ and tRNA^Ser(UCN)^, respectively. The samples from *TRUB1^KO^* (KO1) and WT cell lines were deacylated (DA) by heating for 10 min at 60°C at pH 8.3, electrophoresed, and hybridized with DIG-labeled oligonucleotide probes as described above.

To further assess if the TRUB1 deficiency alters the tRNA metabolism, we subjected mitochondrial RNAs from two *TRUB1*^KO^ cell lines or those with overexpression of *TRUB1* cDNA and HeLa cell lines to Northern blot with DIG-labeled oligodeoxynucleotide probes for tRNA^Asn^, tRNA^Gln^, tRNA^Glu^, tRNA^Pro^, tRNA^Ser(UCN)^ as representatives of the whole L-strand transcription unit and tRNA^Leu(UUR)^, tRNA^Ser(AGY)^, tRNA^Leu(CUN)^, tRNA^Met^ derived from the H-strand transcription unit, as well as a nucleus-encoded mitochondrial 5S RNA (under denaturing condition) (44,45). As shown in Figure 6A and supplemental Figure 4, the amount of these tRNAs in two *TRUB1*^KO^ cell lines were comparable with those in the HeLa cells and *TRUB1*^KO^ cell lines expressing *TRUB1* cDNA.

To understand the effect of *TRUB1* deletion on the aminoacylation of tRNAs, we assessed the aminoacylation properties of tRNA^Asn^, tRNA^Gln^, tRNA^Glu^, tRNA^Pro^, tRNA^Met^, tRNA^Leu(UUR)^, tRNA^Ser(UCN)^ and tRNA^Thr^ by the use of electrophoresis in an acidic polyacrylamide/urea gel system to separate uncharged tRNA species from the corresponding charged tRNA,electroblotting and hybridizing with tRNA probes described above (46,47). To further distinguish nonaminoacylated tRNA from aminoacylated tRNA, samples of tRNAs were deacylated by being heated for 10 min at 60^o^C at pH 8.3 and then run in parallel. As shown in Figure 6B, the upper and lower bands represented the charged and uncharged tRNA, respectively. Despite not well-separated charged and uncharged tRNA^Asn^, tRNA^Gln^, tRNA^Glu^, tRNA^Pro^ and tRNA^Ser(UCN)^ in the acidic urea PAGE system as described previously (46,47), there were no obvious differences in electrophoretic mobility and aminoacylation levels of tRNA^Asn^, tRNA^Gln^, tRNA^Glu^, tRNA^Pro^ and tRNA^Ser(UCN)^ as well as tRNA^Met^, tRNA^Leu(UUR)^ and tRNA^Thr^ between *TRUB1^KO^* cells and WT HeLa cells. These data indicated the TRUB1 deficiency may not affect the aminoacylation properties of mitochondrial tRNAs.

### Impairment of mitochondrial translation

In order to investigate whether the absence of TRUB1 impaired the OXPHOS biogenesis, we carried out the Western blot analysis to examine the levels in 18 subunits of OXPHOS complexes in *TRUB1^KO^* and WT HeLa cells using TOM20 or GAPDH as loading control. These subunits included 8 mtDNA-encoding proteins (ND1, ND3, ND4, ND4L, ND5, CYTB, CO2 and ATP8), 10 nucleus-encoding polypeptides: NDUFA1, NDUFS1 and NDUFS1 [subunits of NADH:ubiquinone oxidoreductase (complex I)], SDHB [subunits of succinate ubiquinone oxidoreductase (complex II)], UQCRC2 and UQCRFS1 [subunits of ubiquinol-cytochrome c reductase (complex III)], COXIV and COX17 [subunits of cytochrome *c* oxidase (complex IV)], ATP5B and ATP5F1 [subunits of H^+^-ATPase (complex V)] (52).

**Figure 7.**
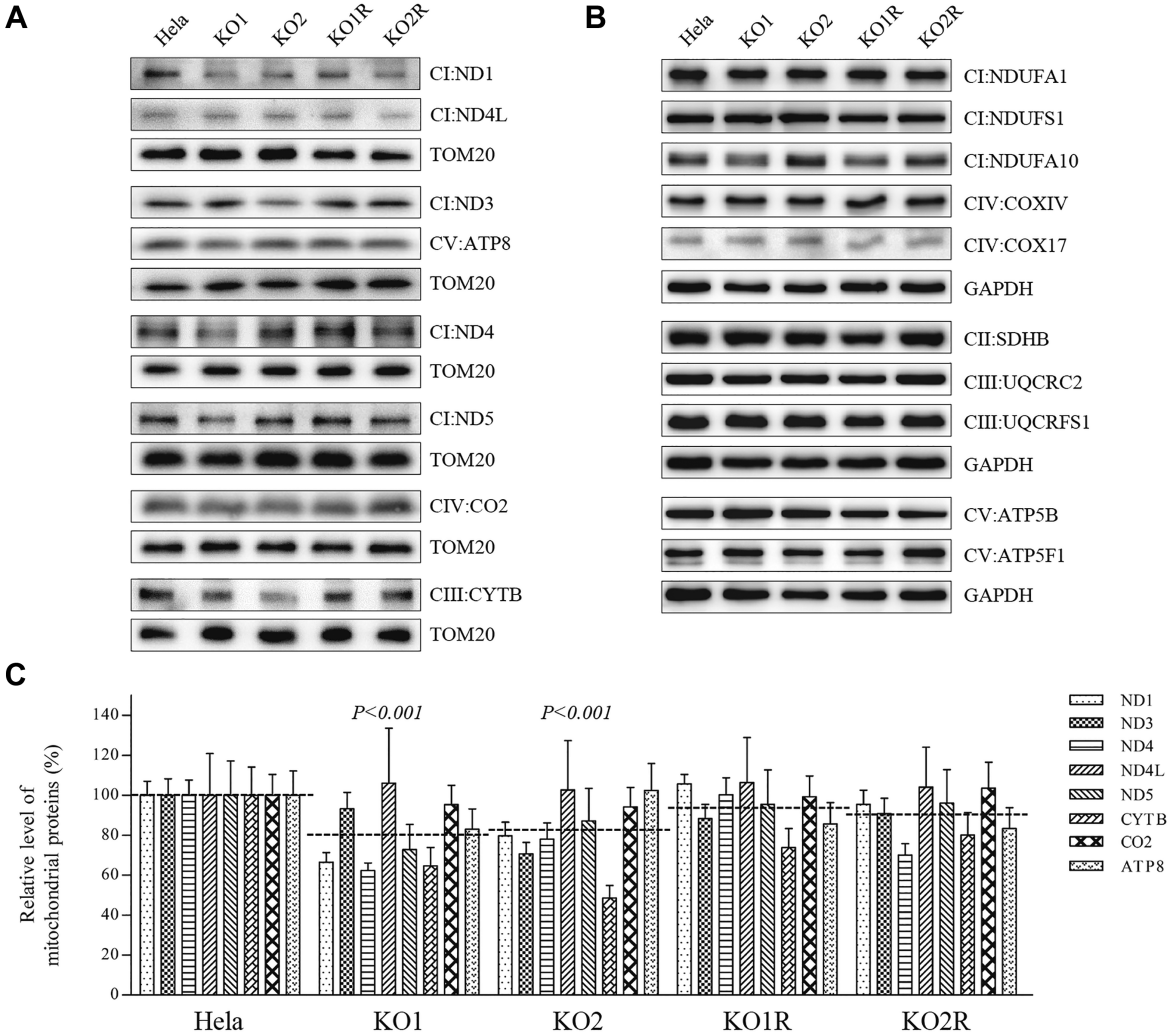
Western blot analysis of mitochondrial proteins. Twenty micrograms of total cellular proteins from various cell lines was electrophoresed through a denaturing polyacrylamide gel, electroblotted and hybridized with antibodies for (**A**) 8 mtDNA-encoding (ND1, ND3, ND4, ND4L, ND5, CYTB, CO2 and ATP8) and (**B**) 10 nucleus-encoding (NDUFA1, NDUFS1, NDUFA10, SDHB, UQCRC2, UQCRFS1, COXIV, COX17, ATP5B and ATP5F1) subunits of OXPHOS (CI, CII, CIII, CIV and CV represented complexes I, II, III, IV and V, respetively), TOM20 or GAPDH as a loading control, respectively. (**C**) Quantification of mitochondrial protein levels. Average relative ND1, ND3, ND4, ND4L, ND5, CYTB, CO2, and ATP8 content per cell, normalized to the average content per cell of TOM20 in *TRUB1*^KO^ and WT cell lines. The values for the *TRUB1^KO^* cell lines are expressed as percentages of the values for the WT cell line. The error bars indicate two standard errors of the means, the horizontal dashed lines represent the average value for each group. The calculations were based on three independent determinations in each cell line. *P* indicates the significance, according to the *t*-test, of the differences between *TRUB1^KO^* and WT cell lines.

As shown in Figure 7A, the various decreases in the levels of 8 mtDNA-encoding proteins (but not of ND4L, CO2 and ATP8) were observed in *TRUB1^KO^* cell lines, as compared with these in the WT cell line. As shown in Figure 7B, the levels of ND1, ND3, ND4, ND4L, ND5, CYTB, CO2 and ATP8 were 66%, 93%, 62%, 106%, 73%, 65%, 95% and 83%, with an average of 81% (*P* < 0.001) in *TRUB1*^KO1^ cells, and 79%, 70%, 78%, 102%, 87%, 49%, 94% and 102%, with an average of 83% (*P <* 0.001) in *TRUB1*^KO2^ cell line, relative to the mean values measured in the WT cell line. However, the overexpression of *TRUB1* elevated the levels of these subunits.

As shown in Figure 7C, the various increases in the levels of 10 nucleus-encoding subunits (but not of UQCRC2) were measured in in *TRUB1*^KO^ cell lines, as compared with WT cell line. As shown in supplemental Figure 5, the levels of NDUFA1, NDUFS1, NDUFA10, SDHB, UQCRC2, UQCRFS1, COXIV, COX17, ATP5B and ATP5F1 were 120%, 116%, 104%, 117%, 95%, 110%, 126%, 141%, 125% and 124%, with an average of 118% (*P* < 0.001) in *TRUB1*^KO1^ cell line, and 121%, 123%, 126%, 112%, 99%, 112%,116%, 133%, 120% and 117%, with an average of 118% (*P* < 0.001) in *TRUB1*^KO2^ cell line, relative to the mean values measured in the WT cell line. However, the levels of these proteins were reduced by the overexpression of *TRUB1* cDNA. These indicated the impact of TRUB1 on mitochondrial translation.

### Defective assembly and activity of OXPHOS complexes

**Figure 8.**
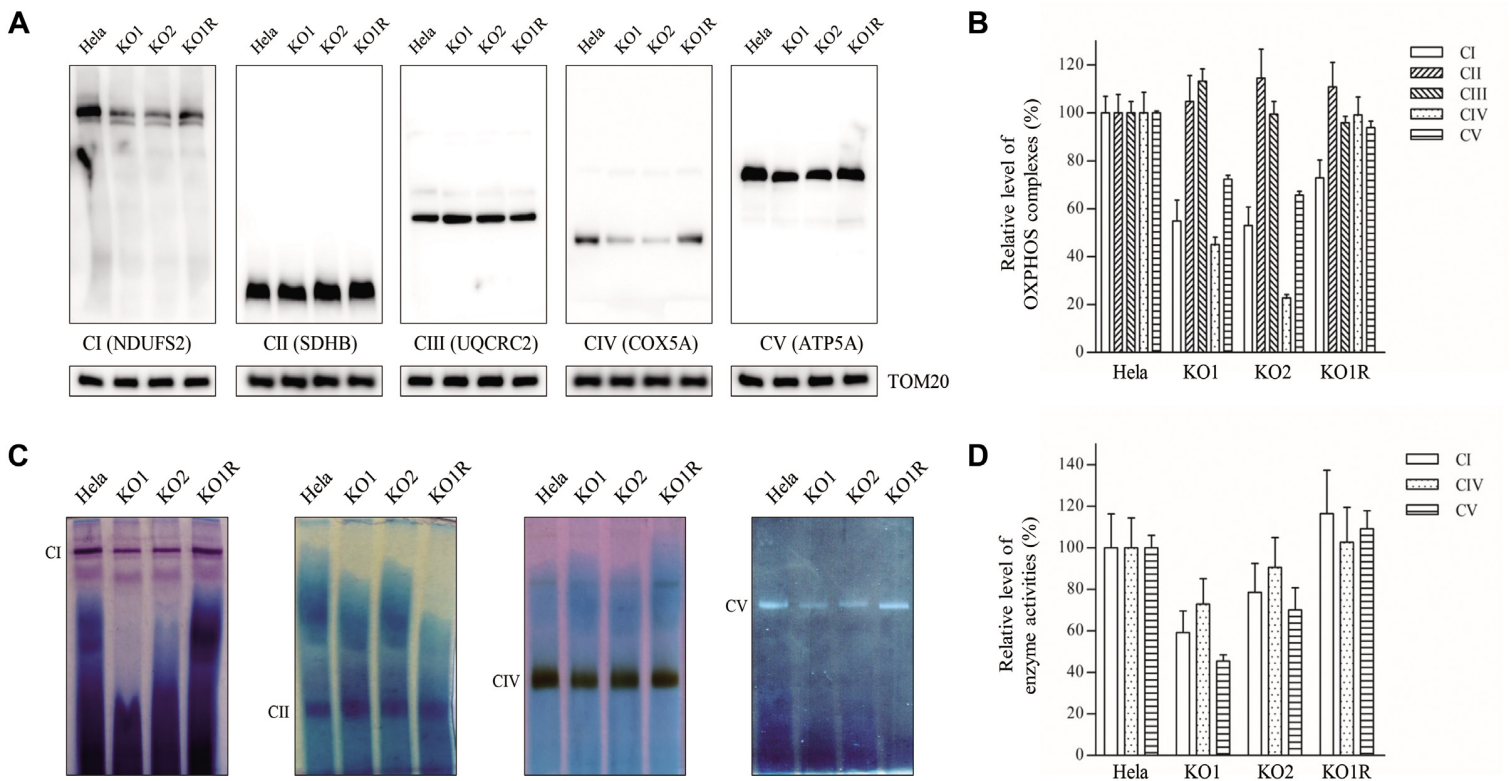
Analysis of OXPHOS complexes. (**A**) The steady-state levels of five OXPHOS complexes by Blue-Native gel electrophoresis. Twenty micrograms of mitochondrial proteins from various cell lines were electrophoresed through a Blue-Native gel, electroblotted and hybridized with antibodies specific for subunits of five OXPHOS complexes (NDUFS2 antibody for complex I, SDHB antibody for complex II, UQCRC2 antibody for complex III, COX5A antibody for complex IV and ATP5A antibody for complex V), and with TOM20 as a loading control. (**B**) Quantification of levels of complexes I, II, III, IV and V in mutant and WT cell lines. The calculations were based on three independent experiments. (**C**) In-gel activity of complexes I, II, IV and V. The activities of OXPHOS complexes from various cell lines after BN-PAGE were measured in the presence of specific substrates [NADH and NTB for complex I, sodium succinate, phenazine methosulfate, and NTB for complex II, DAB and cytochrome c for complex IV, glycine, MgSO_4_, ATP and Pb(NO_3_)_2_ for complex V]. (**D**) Quantification of in-gel activities of complexes I, IV and V. The calculations were based on three independent determinations in each cell line. Graph details and symbols are explained in the legend to Figure 7.

We examined the consequence of the TRUB1 deficiency on the assembly and activities of OXPHOS complexes. Mitochondria isolated from various cell lines were analyzed by BN-PAGE and western blot analysis (49,50,53). As shown in Figure 8A, *TRUB1*^KO^ cell lines exhibited aberrant assembly of complex I, IV and V. In particular, the levels of complex I, II, III, IV and V were 55%, 105%, 113%, 45% and 72% in *TRUB1*^KO1^ cell line, and 53%, 114%, 99%, 23% and 65% in *TRUB1*^KO2^ cell line, relative to the mean values measured in the WT cell line, respectively (Figure 8B).

We then analyzed the stability and activities of complexes I, II, IV and V using the in-gel activity assay. Mitochondrial membrane proteins isolated from various cell lines were separated by BN-PAGE and stained with specific substrates of complexes I, II, IV and V (50,53). Defective assembly of complexes I, IV and V were further confirmed in the *TRUB1*^KO^ cell lines, as compared with WT cell line (Figure 8C and D). In particular, the in-gel activities of complexes I, IV and V in *TRUB1*^KO1^ cell line were 59%, 73% and 45%, and in *TRUB1*^KO2^ cell line were 78%, 91% and 70%, relative to the average values of WT cell line, respectively. In contrast, the in-gel activities of complexes II in the *TRUB1*^KO^ cell lines were comparable with those of WT cell line. Notably, the overexpression of *TRUB1* cDNA restored the defective assembly and activities of OXPHOS caused by TRUB1 deficiency.

## DISCUSSION

Pseudouridine located at position 55 (Ψ55) in the TΨC arm of tRNA is a nearly universally conserved RNA modification found in all three domains of life (1,3). In the present study, we revealed the presence of Ψ55 modification in human mitochondrial tRNA^Asn^, tRNA^Gln^, tRNA^Glu^, tRNA^Pro^, tRNA^Met^, tRNA^Leu(UUR)^ and tRNA^Ser(UCN)^ but not in other 15 mitochondrial tRNAs. In *E. coli*, tRNA pseudouridine 55 synthase (TRUB) catalyzes the pseudouridine formation at U55 by recognizing the T-arm with a 17-base stem–loop structure at position 49–65 (24,54–56). In particular, TRUB recognized a consensus base sequence (U54, U55 and A58) within 7 base T-loop (24). In human, TRUB family included at least three members: TRUB1, TRUB2 and Cbf5/DKC1 sharing highly conserved active site consensus sequences HXGXLD (Supplemental Figure 6). DKC1 primarily functions in ribosomal RNA (rRNA) pseudouridylation, while TRUB2 is involved in mitochondrial mRNA pseudouridylation (29–32). Notably, the tRNA Ψ55 synthase activity of TRUB1 was primarily present in the nucleus (32,33). In this study, both immunofluorescence and cellular fraction assays demonstrated that TRUB1 localizes at mitochondria but not exclusively. These suggested that TRUB1 may play a role in the formation of Ψ55 in tRNAs not only in the nucleus but also mitochondrion, especially in the formation of Ψ55 in mitochondrial tRNA^Asn^, tRNA^Gln^, tRNA^Glu^, tRNA^Pro^, tRNA^Met^, tRNA^Leu(UUR)^ and tRNA^Ser(UCN)^. In this investigation, two *TRUB1*^KO^ cell lines with different alleles generated by with CRISPR/Cas9 system exhibited the complete loss of Ψ55 modification in tRNA^Asn^, tRNA^Gln^, tRNA^Glu^ and tRNA^Pro^. On contrast, the TRUB1 deficiency did not affect the formation of the Ψ55 modification in the mitochondrial tRNA^Met^, tRNA^Leu(UUR)^ and tRNA^Ser(UCN)^, as well as cytosolic tRNA^Thr(ACU)^, tRNA^Met(AUG)^, tRNA^Tyr(UAC)^ and tRNA^His(CAC)^. Furthermore, an *in vitro* enzymatic assay revealed that recombinant human TRUB1 protein indeed catalyzed the efficient formation of Ψ at position 55 in tRNA^Asn^ and tRNA^Gln^, but not in tRNA^Met^ and tRNA^Arg^. Notably, the overexpression of *TRUB1* cDNA reversed the deficient formation of Ψ55 in tRNA^Asn^, tRNA^Gln^, tRNA^Glu^ and tRNA^Pro^ in *TRUB1^KO^* cell lines. These data demonstrated that TRUB1 is responsible for the formation of Ψ55 in mitochondrial tRNA^Asn^, tRNA^Gln^, tRNA^Glu^ and tRNA^Pro^. However, the biosynthesis of Ψ55 in human mitochondrial tRNA^Met^, tRNA^Leu(UUR)^ and tRNA^Ser(UCN)^ may be catalyzed by other pseudouridine synthase(s). In fact, the certain structural requirements may be critical for tRNA pseudouridine synthase activity (55,56). As shown in the Supplemental Table S2, all these 7 tRNAs with Ψ55 modification shared a consensus base sequence (U54, U55 and A58) in the T-loop. The tRNA^Met^ contains 6 base loop, in contrast with the presence of 7 base loop in other 6 tRNAs. Furthermore,the A58 of tRNA^Leu(UUR)^ and tRNA^Ser(UCN)^ are modified to m^1^A58 (6). These discrepancies may be attributed to different substrate recognition by TRUB1 and other pseudouridine synthase(s).

In fact, the Ψ55 in the TΨC arm forms a tertiary base pair with the conserved 18A/G in D-loop and stabilizes the L-shaped tRNA structure (22,23,55,57,58). Thus, the loss of Ψ55 with TRUB1 deficiency destabilized the base-pairing (18A/G-Ψ55) of tRNA^Asn^, tRNA^Gln^, tRNA^Glu^ and tRNA^Pro^, and thereby impacted their structure and function. In fact, the instability of mutant mitochondrial tRNA^Glu^ was evidenced by electrophoretic mobility changes and sensitivity to S1-mediated digestion of tRNA^Glu^ with the loss of Ψ55 caused by m.14692A > G (55U > C) mutation associated with diabetes and deafness (23). In the present study, various electrophoretic mobility changes of tRNA^Gln^, tRNA^Glu^, tRNA^Asn^ and tRNA^Pro^ were observed in *TRUB1*^KO^ cell lines: tRNA^Gln^ and tRNA^Glu^ migrated slower and tRNA^Asn^, tRNA^Pro^ in *TRUB1*^KO^ cells migrated faster than those of wild type cell line. The conformation changes of these tRNAs may be due to the destabilization of the base-pairing (18A/G-Ψ55) of tRNA^Asn^, tRNA^Gln^, tRNA^Glu^ and tRNA^Pro^ and the resultant aberrant structure tertiary structure, caused by the ablation of TRUB1. Furthermore, the tRNA^Gln^, tRNA^Glu^ and tRNA^Pro^ from *TRUB1*^KO^ cell lines were more sensitive to S1-mediated digestion than those from WT cell line. However, there was no effects of *TRUB1* ablation on the steady state levels of tRNA^Asn^, tRNA^Gln^, tRNA^Glu^ and tRNA^Pro^ under denatured conditions, in contrast with the decrease in the steady-state level of tRNA^Glu^ in mutant cell lines carrying the tRNA^Glu^ 14692A > G mutation (23). These discrepancies may be attributed to the different mechanism of pseudouridine deficiency, even though these shared identical pseudouridine modifications at U55 in mitochondrial tRNA^Glu^, tRNA^Gln^, tRNA^Asn^ and tRNA^Pro^. Moreover, the lacks of TRUB1 appeared not to affect the aminoacylation capacity of these mitochondrial tRNAs. Indeed, the TruB-affected Ψ55 modification of tRNA in bacteria was not essential but required for the low temperature adaptation (59,60).

The TRUB1 deficiency-induced failures in tRNA metabolism may impact the mitochondrial translation and the biogenesis of oxidative phosphorylation system, comprised of mtDNA-encoded subunit(s) and nuclear-encoded subunits. These mtDNA-encoded subunits appear to act as seeds for building new complexes, which requires nuclear-encoded subunit import and assembly with the assistance of assembly factors (61). In our previous study, lymphoblastoid cell lines carrying the tRNA^Glu^ 14692A > G (55U > C) mutation exhibited reduced levels of mitochondrial proteins (an average decrease of ∼29%) (23). In the present study, the variable decreases in levels of 8 mtDNA-encoded polypeptides (an average decrease of ∼28%) were observed in the *TRUB1*^KO^ mutant cell lines. In particular, *TRUB1*^KO^ mutant cell lines exhibited marked reductions (44%) in the levels of CYTB, relative mild reductions (20–30%) in the levels of ND1, ND3, ND4 and ND5, but very mild decreases (3–5%) in the levels of ATP8 and CO2. In contrast to what was previously shown in cells carrying the tRNA^Lys^ 8344A > G or tRNA^Ser(UCN)^ 7445A > G mutation (62,63), polypeptides levels in mutant cell lines, relative to those in WT cell lines, did not significantly correlate with the number or density of asparagine, glutamic acid, glutamine, proline codon or these 4 codons (Supplemental Table S3). However, TRUB1 deficiency elevated the expression levels of nucleus-encoded subunits of OXPHOS, in contrast with drastic effects of those subunits observed in the *YARS2* knockout cell lines (35). This could be due to a compensatory response to impaired synthesis of mtDNA encoded polypeptides (50,64). The impaired synthesis of mtDNA encoding subunits of OXPHOS gave rise to aberrant assembly and instability of complexes I, IV and V observed in the *TRUB1*^KO^ cell lines by BN-PAGE and Western blot assays. As a consequence, these defects yielded the reduced activities of these respiratory chain enzyme complexes. In-gel activities assays revealed that *TRUB1*^KO^ cell lines exhibited the significant decreases in the activities of complexes I, IV and V, as compared with those in WT cell line. These data highlight the critical role of tRNA modification failures in producing mitochondrial dysfunctions, as in the cases of cell lines carrying the tRNA^Glu^ 14692A > G, tRNA^Asp^ 7551A > G, tRNA^Met^ 4435A > G, tRNA^Ile^ 4295A > G mutations, *TRMU* and *TRMT5* mutations (8,15,20,23,65). The defetive oxidative phosphorylation may result in the subsequent failure of cellular energetic processes.

In summary, our data highlighted the presence of Ψ55 in mitochondrial tRNA^Asn^, tRNA^Gln^, tRNA^Glu^, tRNA^Pro^, tRNA^Met^, tRNA^Leu(UUR)^ and tRNA^Ser(UCN)^. Our findings demonstrated that the TRUB1 is responsible for the formation of Ψ55 in mitochondrial tRNA^Asn^, tRNA^Gln^, tRNA^Glu^ and tRNA^Pro^ and impacts on mitochondrial tRNA metabolism, translation and the biogenesis of oxidative phosphorylation system.

## DATA AVAILABILITY

The authors declare that [the/all other] data supporting the findings of this study are available within the article [and its supplementary information files].

## Supplementary Material

gkac698_Supplemental_File

## References

[1] Phizicky E.M. , Hopper A.K. tRNA biology charges to the front. Genes Dev. 2010; 24:1832–1860. 20810645 10.1101/gad.1956510 PMC2932967

[2] Suzuki T. , Nagao A., Suzuki T. Human mitochondrial tRNAs: biogenesis, function, structural aspects, and diseases. Annu. Rev. Genet. 2011; 45:299–329. 21910628 10.1146/annurev-genet-110410-132531

[3] El Yacoubi B. , Bailly M., de Crécy-Lagard V. Biosynthesis and function of posttranscriptional modifications of transfer RNAs. Annu. Rev. Genet. 2012; 46:69–95. 22905870 10.1146/annurev-genet-110711-155641

[4] Suzuki T. , Suzuki T. A complete landscape of post-transcriptional modifications in mammalian mitochondrial tRNAs. Nucleic Acids Res. 2014; 42:7346–7357. 24831542 10.1093/nar/gku390 PMC4066797

[5] Boccaletto P. , Machnicka M.A., Purta E., Piatkowski P., Baginski B., Wirecki T.K., de Crecy-Lagard V., Ross R., Limbach P.A., Kotter A. et al. MODOMICS: a database of RNA modification pathways. 2017 update. Nucleic Acids Res. 2018; 46:D303–D307. 29106616 10.1093/nar/gkx1030 PMC5753262

[6] Suzuki T. , Yashiro Y., Kikuchi I., Ishigami Y., Saito H., Matsuzawa I., Okada S., Mito M., Iwasaki S., Ma D. et al. Complete chemical structures of human mitochondrial tRNAs. Nat. Commun. 2020; 11:4269. 32859890 10.1038/s41467-020-18068-6 PMC7455718

[7] Agris P.F. , Vendeix F.A., Graham W.D. tRNA’s wobble decoding of the genome: 40 years of modification. J. Mol. Biol. 2007; 366:1–13. 17187822 10.1016/j.jmb.2006.11.046

[8] Wang M. , Peng Y., Zheng J., Zheng B., Jin X., Liu H., Wang Y., Tang X., Huang T., Jiang P. et al. A deafness-associated tRNA^Asp^ mutation alters the m^1^G37 modification, aminoacylation and stability of tRNA^Asp^ and mitochondrial function. Nucleic Acids Res. 2016; 44:10974–10985. 27536005 10.1093/nar/gkw726 PMC5159531

[9] Agris P.F. The importance of being modified: roles of modified nucleosides and mg^2+^ in RNA structure and function. Prog. Nucleic Acid Res. Mol. Biol. 1996; 53:79–129. 8650309 10.1016/s0079-6603(08)60143-9

[10] Allner O. , Nilsson L. Nucleotide modifications and tRNA anticodon-mRNA codon interactions on the ribosome. RNA. 2011; 17:2177–2188. 22028366 10.1261/rna.029231.111 PMC3222130

[11] Johansson M.J. , Esberg A., Huang B., Bjork G.R., Bystrom A.S. Eukaryotic wobble uridine modifications promote a functionally redundant decoding system. Mol. Cell. Biol. 2008; 28:3301–3312. 18332122 10.1128/MCB.01542-07 PMC2423140

[12] Kirino Y. , Goto Y., Campos Y., Arenas J., Suzuki T. Specific correlation between the wobble modification deficiency in mutant tRNAs and the clinical features of a human mitochondrial disease. Proc. Natl. Acad. Sci. U.S.A. 2005; 102:7127–7132. 15870203 10.1073/pnas.0500563102 PMC1129107

[13] Hori H. Methylated nucleosides in tRNA and tRNA methyltransferases. Front. Genet. 2014; 5:144. 24904644 10.3389/fgene.2014.00144 PMC4033218

[14] de Crécy-Lagard V. , Boccaletto P., Mangleburg C.G., Sharma P., Lowe T.M., Leidel S.A., Bujnicki J.M. Matching tRNA modifications in humans to their known and predicted enzymes. Nucleic Acids Res. 2019; 47:2143–2159. 30698754 10.1093/nar/gkz011 PMC6412123

[15] Guan M.X. , Yan Q., Li X., Bykhovskaya Y., Gallo-Teran J., Hajek P., Umeda N., Zhao H., Garrido G., Mengesha E. et al. Mutation in TRMU related to transfer RNA modification modulates the phenotypic expression of the deafness-associated mitochondrial 12S ribosomal RNA mutations. Am. J. Hum. Genet. 2006; 79:291–302. 16826519 10.1086/506389 PMC1559489

[16] Umeda N. , Suzuki T., Yukawa M., Ohya Y., Shindo H., Watanabe K., Suzuki T. Mitochondria-specific RNA-modifying enzymes responsible for the biosynthesis of the wobble base in mitochondrial tRNAs. Implications for the molecular pathogenesis of human mitochondrial diseases. J. Biol. Chem. 2005; 280:1613–1624. 15509579 10.1074/jbc.M409306200

[17] Wang X. , Yan Q., Guan M.X. Combination of the loss of cmnm^5^U34 with the lack of s^2^U34 modifications of tRNA^Lys^, tRNA^Glu^, and tRNA^Gln^ altered mitochondrial biogenesis and respiration. J. Mol. Biol. 2010; 395:1038–1048. 20004207 10.1016/j.jmb.2009.12.002 PMC2818684

[18] Chen D. , Zhang Z., Chen C., Yao S., Yang Q., Li F., He X., Ai C., Wang M., Guan M.X. Deletion of gtpbp3 in zebrafish revealed the hypertrophic cardiomyopathy manifested by aberrant mitochondrial tRNA metabolism. Nucleic Acids Res. 2019; 47:5341–5355. 30916346 10.1093/nar/gkz218 PMC6547414

[19] Yarham J.W. , Lamichhane T.N., Pyle A., Mattijssen S., Baruffini E., Bruni F., Donnini C., Vassilev A., He L., Blakely E.L. et al. Defective i^6^A37 modification of mitochondrial and cytosolic tRNAs results from pathogenic mutations in TRIT1 and its substrate tRNA. PLoS Genet. 2014; 10:e1004424. 24901367 10.1371/journal.pgen.1004424 PMC4046958

[20] Powell C.A. , Kopajtich R., D'Souza A.R., Rorbach J., Kremer L.S., Husain R.A., Dallabona C., Donnini C., Alston C.L., Griffin H et al. TRMT5 mutations cause a defect in post-transcriptional modification of mitochondrial tRNA associated with multiple respiratory-chain deficiencies. Am. J. Hum. Genet. 2015; 97:319–328. 26189817 10.1016/j.ajhg.2015.06.011 PMC4573257

[21] Helm M. , Brulé H., Degoul F., Cepanec C., Lerouxm J.P., Giegé R., Florentz C. The presence of modified nucleotides is required for cloverleaf folding of a human mitochondrial tRNA. Nucleic Acids Res. 1998; 26:1636–1643. 9512533 10.1093/nar/26.7.1636 PMC147479

[22] Roovers M. , Hale C., Tricot C., Terns M.P., Terns R.M., Grosjean H., Droogmans L. Formation of the conserved pseudouridine at position 55 in archaeal tRNA. Nucleic Acids Res. 2006; 34:4293–4301. 16920741 10.1093/nar/gkl530 PMC1616971

[23] Wang M. , Liu H., Zheng J., Chen B., Zhou M., Fan W., Wang H., Liang X., Zhou X., Eriani G. et al. A deafness- and diabetes-associated tRNA mutation causes deficient pseudouridinylation at position 55 in tRNA^Glu^ and mitochondrial dysfunction. J. Biol. Chem. 2016; 291:21029–21041. 27519417 10.1074/jbc.M116.739482 PMC5076513

[24] Gu X. , Yu M., Ivanetich K.M., Santi DV. Molecular recognition of tRNA by tRNA pseudouridine 55 synthase. Biochemistry. 1998; 37:339–343. 9425055 10.1021/bi971590p

[25] Becker H.F. , Motorin Y., Planta R.J., Grosjean H. The yeast gene YNL292w encodes a pseudouridine synthase (Pus4) catalyzing the formation of psi55 in both mitochondrial and cytoplasmic tRNAs. Nucleic Acids Res. 1997; 25:4493–4499. 9358157 10.1093/nar/25.22.4493 PMC147073

[26] Keffer-Wilkes L.C. , Veerareddygari G.R., Kothe U. RNA modification enzyme TruB is a tRNA chaperone. Proc. Natl. Acad. Sci. U.S.A. 2016; 113:14306–14311. 27849601 10.1073/pnas.1607512113 PMC5167154

[27] Hamma T. , Ferré-D’Amaré A.R. Pseudouridine synthases. Chem. Biol. 2006; 13:1125–1135. 17113994 10.1016/j.chembiol.2006.09.009

[28] Zucchini C. , Strippoli P., Biolchi A., Solmi R., Lenzi L., D’Addabbo P., Carinci P., Valvassori L. The human TruB family of pseudouridine synthase genes, including the dyskeratosis congenita 1 gene and the novel member *TRUB1*. Int. J. Mol. Med. 2003; 11:697–704. 12736709

[29] Garus A. , Autexier C. Dyskerin: an essential pseudouridine synthase with multifaceted roles in ribosome biogenesis, splicing, and telomere maintenance. RNA. 2021; 27:1441–1458. 34556550 10.1261/rna.078953.121 PMC8594475

[30] Antonicka H. , Choquet K., Lin Z.Y., Gingras A.C., Kleinman C.L., Shoubridge E.A. A pseudouridine synthase module is essential for mitochondrial protein synthesis and cell viability. EMBO Rep. 2017; 18:28–38. 27974379 10.15252/embr.201643391 PMC5210091

[31] Arroyo J.D. , Jourdain A.A., Calvo S.E., Ballarano C.A., Doench J.G., Root D.E., Mootha V.K. A genome-wide CRISPR death screen identifies genes essential for oxidative phosphorylation. Cell Metab. 2016; 24:875–885. 27667664 10.1016/j.cmet.2016.08.017 PMC5474757

[32] Mukhopadhyay S. , Deogharia M., Gupta R. Mammalian nuclear TRUB1, mitochondrial TRUB2, and cytoplasmic PUS10 produce conserved pseudouridine 55 in different sets of tRNA. RNA. 2021; 27:66–79. 33023933 10.1261/rna.076810.120 PMC7749629

[33] Safra M. , Nir R., Farouq D., Vainberg Slutskin I., Schwartz S. TRUB1 is the predominant pseudouridine synthase acting on mammalian mRNA via a predictable and conserved code. Genome Res. 2017; 27:393–406. 28073919 10.1101/gr.207613.116 PMC5340967

[34] Ofengand J. , Del Campo M., Kaya Y. Mapping pseudouridines in RNA molecules. Methods. 2001; 25:365–373. 11860291 10.1006/meth.2001.1249

[35] Shalem O. , Sanjana N.E., Hartenian E., Shi X., Scott D.A., Mikkelson T., Heckl D., Ebert B.L., Root D.E., Doench J.G. et al. Genome-scale CRISPR-Cas9 knockout screening in human cells. Science. 2014; 343:84–87. 24336571 10.1126/science.1247005 PMC4089965

[36] Yu J. , Liang X., Ji Y., Ai C., Liu J., Zhu L., Nie Z., Jin X., Wang C., Zhang J. et al. PRICKLE3 linked to ATPase biogenesis manifested leber's hereditary optic neuropathy. J. Clin. Invest. 2020; 130:4935–4946. 32516135 10.1172/JCI134965 PMC7456240

[37] Jin X. , Zhang Z., Nie Z., Wang C., Meng F., Yi Q., Chen M., Sun J., Zou J., Jiang P. et al. An animal model for mitochondrial tyrosyl-tRNA synthetase deficiency reveals links between oxidative phosphorylation and retinal function. J. Biol. Chem. 2021; 296:100437. 33610547 10.1016/j.jbc.2021.100437 PMC8010715

[38] Rodina A. , Wang T., Yan P., Gomes E.D., Dunphy M.P., Pillarsetty N., Koren J., Gerecitano J.F., Taldone T., Zong H. et al. The epichaperome is an integrated chaperome network that facilitates tumour survival. Nature. 2016; 538:397–401. 27706135 10.1038/nature19807 PMC5283383

[39] Gong S. , Peng Y., Jiang P., Wang M., Fan M., Wang X., Zhou H., Li H., Yan Q., Huang T. et al. A deafness-associated tRNA^His^ mutation alters the mitochondrial function, ROS production and membrane potential. Nucleic Acids Res. 2014; 42:8039–8048. 24920829 10.1093/nar/gku466 PMC4081083

[40] Jia Z. , Zhang Y., Li Q., Ye Z., Liu Y., Fu C., Cang X., Wang M., Guan M.X. A coronary artery disease-associated tRNA^Thr^ mutation altered mitochondrial function, apoptosis and angiogenesis. Nucleic Acids Res. 2019; 47:2056–2074. 30541130 10.1093/nar/gky1241 PMC6393294

[41] Guo F. , Zhu G. Presence and removal of a contaminating NADH oxidation activity in recombinant maltose-binding protein fusion proteins expressed in escherichia coli. BioTechniques. 2012; 52:247–253. 22482440 10.2144/0000113822

[42] King M.P. , Attardi G. Post-transcriptional regulation of the steady-state levels of mitochondrial tRNAs in hela cells. J. Biol. Chem. 1993; 268:10228–10237. 7683672

[43] Fan W. , Zheng J., Kong W., Cui L., Aishanjiang M., Yi Q., Wang M., Cang X., Tang X., Chen Y. et al. Contribution of a mitochondrial tyrosyl-tRNA synthetase mutation to the phenotypic expression of the deafness-associated tRNA ser(ucn) 7511A>G mutation. J. Biol. Chem. 2019; 294:19292–19305. 31685661 10.1074/jbc.RA119.010598 PMC6916496

[44] Xiao Y. , Wang M., He Q., Xu L., Zhang Q., Meng F., Jia Z., Zhang F., Wang H., Guan M.X. Asymmetrical effects of deafness-associated mitochondrial DNA 7516delA mutation on the processing of RNAs in the H-strand and L-strand polycistronic transcripts. Nucleic Acids Res. 2020; 48:11113–11129. 33045734 10.1093/nar/gkaa860 PMC7641755

[45] Zhao X. , Cui L., Xiao Y., Mao Q., Aishanjiang M., Kong W., Liu Y., Chen H., Hong F., Jia Z. et al. Hypertension-associated mitochondrial DNA 4401A>G mutation caused the aberrant processing of tRNA^Met^, all 8 tRNAs and ND6 mRNA in the light-strand transcript. Nucleic Acids Res. 2019; 47:10340–10356. 31504769 10.1093/nar/gkz742 PMC6821173

[46] Zhou M. , Xue L., Chen Y., Li H., He Q., Wang B., Meng F., Wang M., Guan M.X. A hypertension-associated mitochondrial DNA mutation introduces an m^1^G37 modification into tRNA^Met^, altering its structure and function. J. Biol. Chem. 2018; 293:1425–1438. 29222331 10.1074/jbc.RA117.000317 PMC5787817

[47] Enriquez J.A. , Attardi G. Analysis of aminoacylation of human mitochondrial tRNAs. Methods Enzymol. 1996; 264:183–196. 8965692 10.1016/s0076-6879(96)64019-1

[48] Zhang Q. , He X., Yao S., Lin T., Zhang L., Chen D., Chen C., Yang Q., Li F., Zhu Y.M. et al. Ablation of mto1 in zebrafish exhibited hypertrophic cardiomyopathy manifested by mitochondrion RNA maturation deficiency. Nucleic Acids Res. 2021; 49:4689–4704. 33836087 10.1093/nar/gkab228 PMC8096277

[49] Jha P. , Wang X., Auwerx J. Analysis of mitochondrial respiratory chain super-complexes using blue native polyacrylamide gel electrophoresis (BN-PAGE). Curr. Protoc. Mouse Biol. 2016; 6:1–14. 26928661 10.1002/9780470942390.mo150182 PMC4823378

[50] Fan W. , Jin X., Xu M., Xi Y., Lu W., Yang X., Guan M.X., Ge W. FARS2 deficiency in *Drosophila* reveals the developmental delay and seizure manifested by aberrant mitochondrial tRNA metabolism. Nucleic Acids Res. 2021; 49:13108–13121. 34878141 10.1093/nar/gkab1187 PMC8682739

[51] Claros M.G. , Vincens P. Computational method to predict mitochondrially imported proteins and their targeting sequences. Eur. J. Biochem. 1996; 241:779–786. 8944766 10.1111/j.1432-1033.1996.00779.x

[52] Wallace D.C. Mitochondrial genetic medicine. Nat. Genet. 2018; 50:1642–1649. 30374071 10.1038/s41588-018-0264-z

[53] Vizarra E. , Zeviani M. Blue-native electrophoresis to study the OXPHOS complexes. Methods Mol. Biol. 2021; 2192:287–311. 33230780 10.1007/978-1-0716-0834-0_20

[54] Pan H. , Agarwalla S., Moustakas D.T., Finer-Moore J., Stroud R.M. Structure of tRNA pseudouridine synthase TruB and its RNA complex: RNA recognition through a combination of rigid docking and induced fit. Proc. Natl. Acad. Sci. U.S.A. 2003; 100:12648–12653. 14566049 10.1073/pnas.2135585100 PMC240672

[55] Hoang C. , Ferré-D’Amaré A.R. Cocrystal structure of a tRNA psi55 pseudouridine synthase: nucleotide flipping by an RNA-modifying enzyme. Cell. 2001; 107:929–939. 11779468 10.1016/s0092-8674(01)00618-3

[56] Deogharia M. , Mukhopadhyay S., Joardar A., Gupta R. The human ortholog of archaeal pus10 produces pseudouridine 54 in select tRNAs where its recognition sequence contains a modified residue. RNA. 2019; 25:336–351. 30530625 10.1261/rna.068114.118 PMC6380271

[57] Kim S.H. , Sussman J.L., Suddath F.L., Quigley G.J., McPherson A., Wang A.H., Seeman N.C., Rich A. The general structure of transfer RNA molecules. Proc. Natl. Acad. Sci. U.S.A. 1974; 71:4970–4974. 4612535 10.1073/pnas.71.12.4970 PMC434021

[58] Sekine S. , Nureki O., Shimada A., Vassylyev D.G., Yokoyama S. Structural basis for anticodon recognition by discriminating glutamyl-tRNA synthetase. Nat. Struct. Biol. 2001; 8:203–2066. 11224561 10.1038/84927

[59] Ishida K. , Kunibayashi T., Tomikawa C., Ochi A., Kanai T., Hirata A., Iwashita C., Hiroyuki H. Pseudouridine at position 55 in tRNA controls the contents of other modified nucleotides for low-temperature adaptation in the extreme-thermophilic eubacterium *Thermus**thermophiles*. Nucleic Acids Res. 2011; 39:2304–2318. 21097467 10.1093/nar/gkq1180 PMC3064792

[60] Kinghorn S.M. , O’Byrne C.P., Booth I.R., Stansfield I. Physiological analysis of the role of truB in *Escherichia**coli*: a role for tRNA modification in extreme temperature resistance. Microbiology. 2002; 148:3511–3520. 12427942 10.1099/00221287-148-11-3511

[61] Signes A. , Fernandez-Vizarra E. Assembly of mammalian oxidative phosphorylation complexes I-V and supercomplexes. Essays Biochem. 2018; 62:255–270. 30030361 10.1042/EBC20170098 PMC6056720

[62] Guan M.X. , Enriquez J.A., Fischel-Ghodsian N., Puranam R.S., Lin C.P., Maw M.A., Attardi G. The deafness-associated mitochondrial DNA mutation at position 7445, which affects tRNA^Ser(UCN)^ precursor processing, has long-range effects on NADH dehydrogenase subunit ND6 gene expression. Mol. Cell. Biol. 1998; 18:5868–5879. 9742104 10.1128/mcb.18.10.5868 PMC109173

[63] Enriquez J.A. , Chomyn A., Attardi G. MtDNA mutation in MERRF syndrome causes defective aminoacylation of tRNA^Lys^ and premature translation termination. Nat. Genet. 1995; 10:47–55. 7647790 10.1038/ng0595-47

[64] Wredenberg A. , Lagouge M., Bratic A., Metodiev M.D., Spahr H., Mourier A., Freyer C., Ruzzenente B., Tain L., Gronke S. et al. MTERF3 regulates mitochondrial ribosome biogenesis in invertebrates and mammals. PLoS Genet. 2013; 9:e1003178. 23300484 10.1371/journal.pgen.1003178 PMC3536695

[65] Meng F. , Zhou M., Xiao Y., Mao X., Zheng J., Lin J., Lin T., Ye Z., Cang X., Fu Y. et al. A deafness-associated tRNA mutation caused pleiotropic effects on the m^1^G37 modification, processing, stability and aminoacylation of tRNA^Ile^ and mitochondrial translation. Nucleic Acids Res. 2021; 49:1075–1093. 33398350 10.1093/nar/gkaa1225 PMC7826259

