# Supplementary material for: Human TRUB1 is a highly conserved pseudouridine synthase responsible for the formation of Ψ55 in mitochondrial tRNAAsn, tRNAGln, tRNAGlu and tRNAPro
Source: Nucleic Acids Res. 2022 Aug 26;50(16):9368–81. doi: 10.1093/nar/gkac698 (PMC9458420; doi:10.1093/nar/gkac698)
Supplement: gkac698_Supplemental_File [file gkac698_supplemental_file.pdf]

Supplemental information

Supplemental Figure S1, S2, S3, S4, S5 and S6
Supplemental Table S1, S2 and S3

Supplementary Figure S1

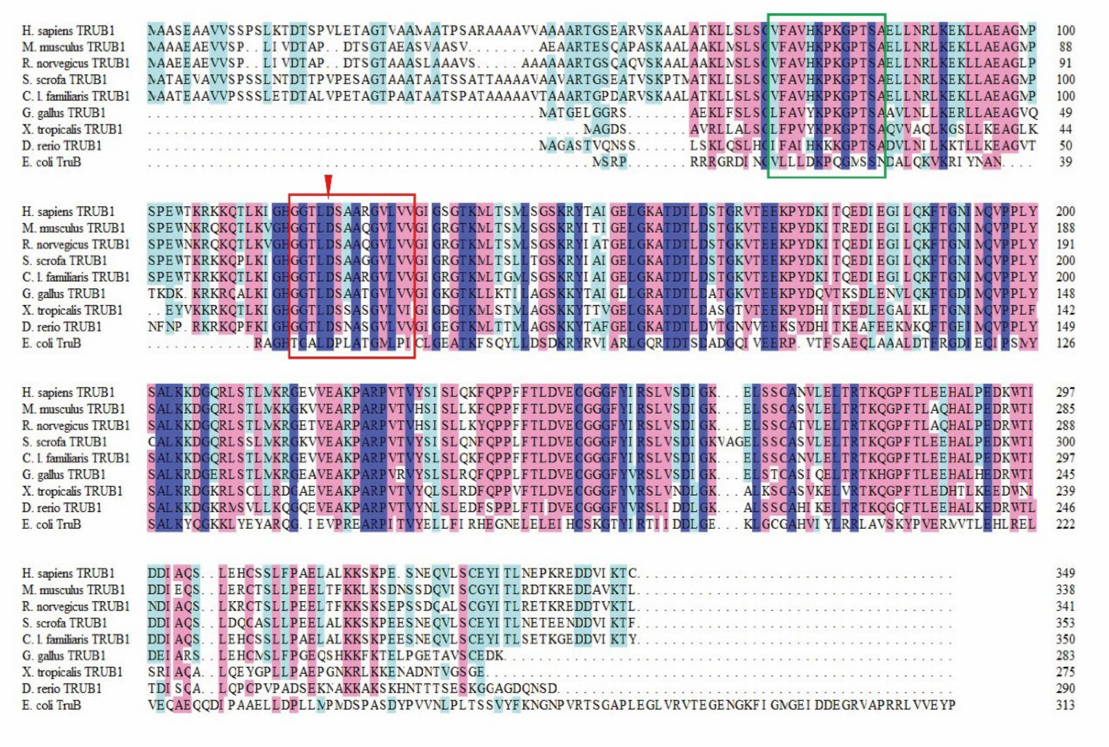

Figure S1. Sequence alignment of human TRUB1 with its homologs. The alignment was generated using the DNAMAN software. The organisms and corresponding accession numbers used for this analysis are as follows: *H. sapiens* (NP_631908.1), *M. musculus* (NP_082391.1), *R. norvegicus* (NP_001012173.1), *S. scrofa* (XP_013839224.1), C. *L. familiaris* (XP_038296608.1), *G. gallus* (XP_421776.1), *X. tropicalis* (XP_002941103.1), *D. rerio* (NP_001122159.1) and *E. coli* (NP_417635.1). Numbers give the position of residues in proteins in relation to the first methionine of the *H. sapiens*. Green box shows the motif I (VFAVHKPKGPTSA box in positions 71-83), the red box shows the motif II (GGTLDS AARGVLVV in positions 117-130) and the red triangle shows the highly conserved 121D residue. Amino acid residues shaded faint blue are identical; those shaded brick red and wathet blue are similar in at least seven residues and five residues of nine homologs, respectively.

Supplementary Figure S2

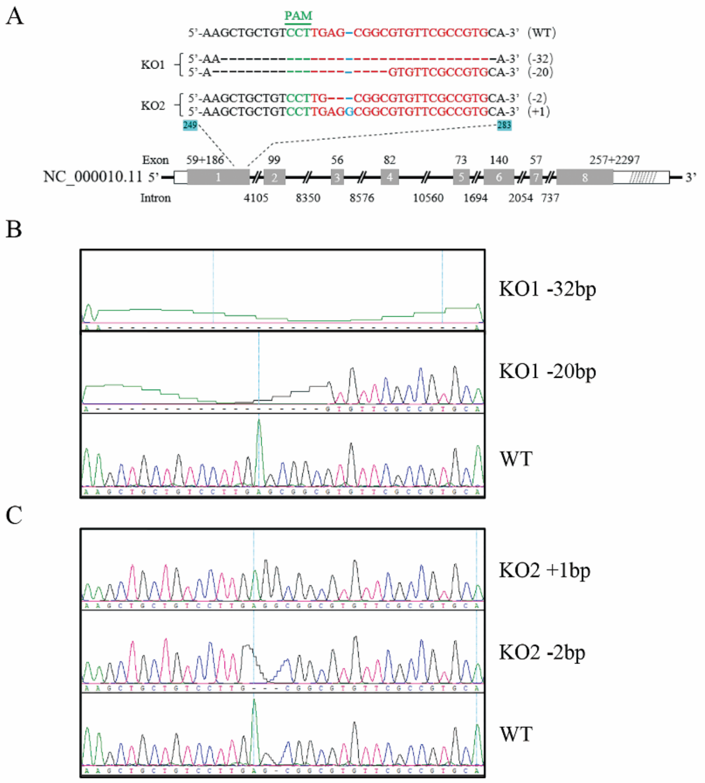

Figure S2. Confirmation of *TRUB1* knockout alleles. (A) Schematic depiction of the human *TRUB1* gene with CRISPR/Cas9 target site at exon 1 as used in this study. Shaded boxes indicate coding regions; open boxes indicate untranslated regions of exons; lines indicate introns. The target sequence of the single guide RNA (sgRNA) is indicated in red. The protospacer adjacent motif (PAM) sequence is indicated in green. Deletions are represented by dashed lines. (B) Partial sequence chromatograms of exon 1 in the *TRUB1* gene in the Hela cell line (WT) and resultant *TRUB1* knock out cell line KO1. (C) Partial sequence chromatograms of exon 1 in the *TRUB1* gene in the Hela cell line (WT) and resultant *TRUB1* knock out cell line KO2.

Supplementary Figure S3

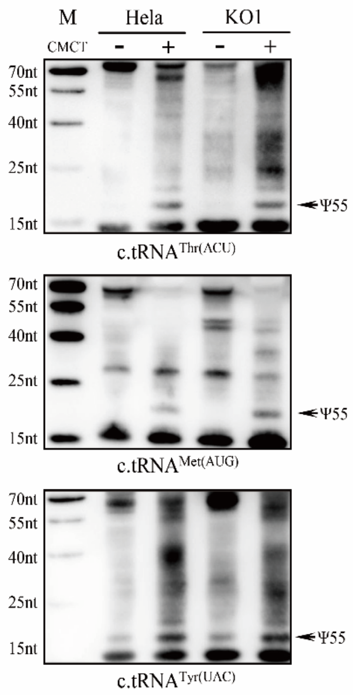

Figure S3. Pseudouridine sequencing of three cytosolic tRNAs. Primer extension analysis of the cytoplasmic $tRNA^{Thr(ACU)}$, $tRNA^{Met(AUG)}$ and $tRNA^{Tyr(UAC)}$. Total RNA was isolated from wild-type (Hela) and *TRUB1* knockout (KO1) cell strains, and treated with (+) or without (-) CMCT, followed by alkali (OH^{-}) treatment. Reverse transcription was carried out using digoxin labeled primers to identify the stops caused by CMC-pseudouridine. The arrow indicates a strong stop at Ψ55. M: marker, DIG-labeled oligonucleotides of variable length.

Supplementary Figure S4

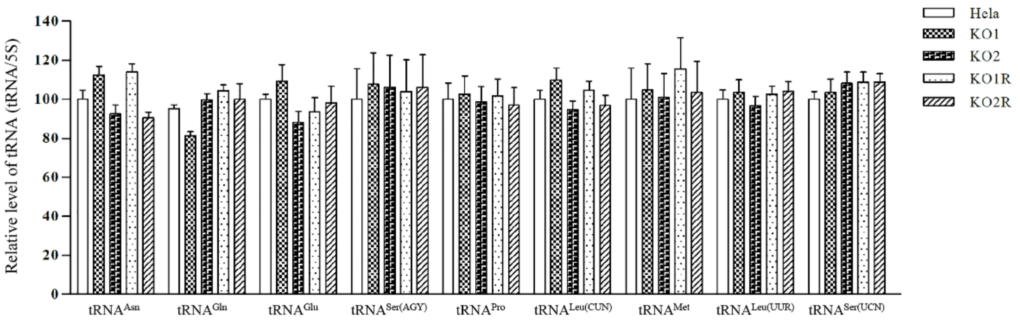

Figure S4. Quantification of the tRNA levels. Average relative each tRNA content per cell was normalized to the average content per cell of 5S rRNA in the wild type and *TRUB1*^{KO} HeLa cell lines. The values for the latter are expressed as percentages of the average values for the wild type Hela cell line. The calculations were based on three independent determination in each cell line. The error bars indicate two standard errors of the mean (SEM).

Supplementary Figure S5

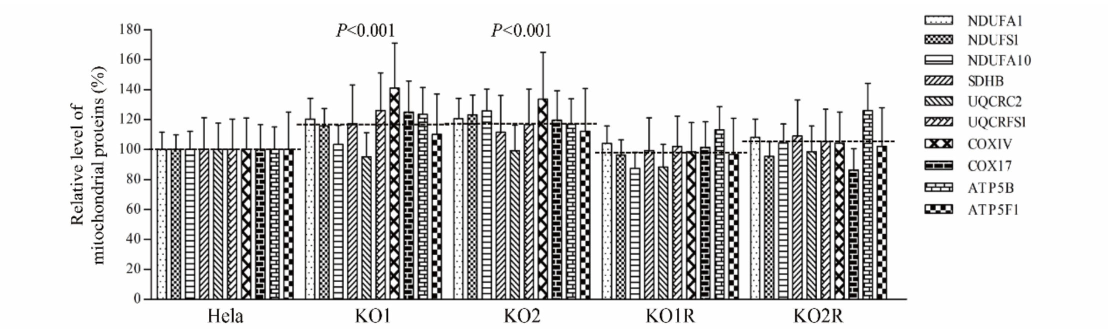

Figure S5. Quantification nucleus-encoding mitochondrial proteins. Average relative NDUFA1, NDUFS1, NDUFA10, SDHB, UQCRC2, UQCRFS1, COXIV, COX17, ATP5B and ATP5F1 content per cell, normalized to the average content per cell of GAPDH in *TRUB1*^{KO} and wildtype Hela cell lines. The value for the knockout cells are expressed as percentages of the values for the control cells. The error bars indicate two standard errors of the means, the horizontal dashed lines represent the average value for each group. P indicates the significance, according to the t-test, of the differences between *TRUB1*^{KO} and control Hela cells.

Supplementary Figure S6

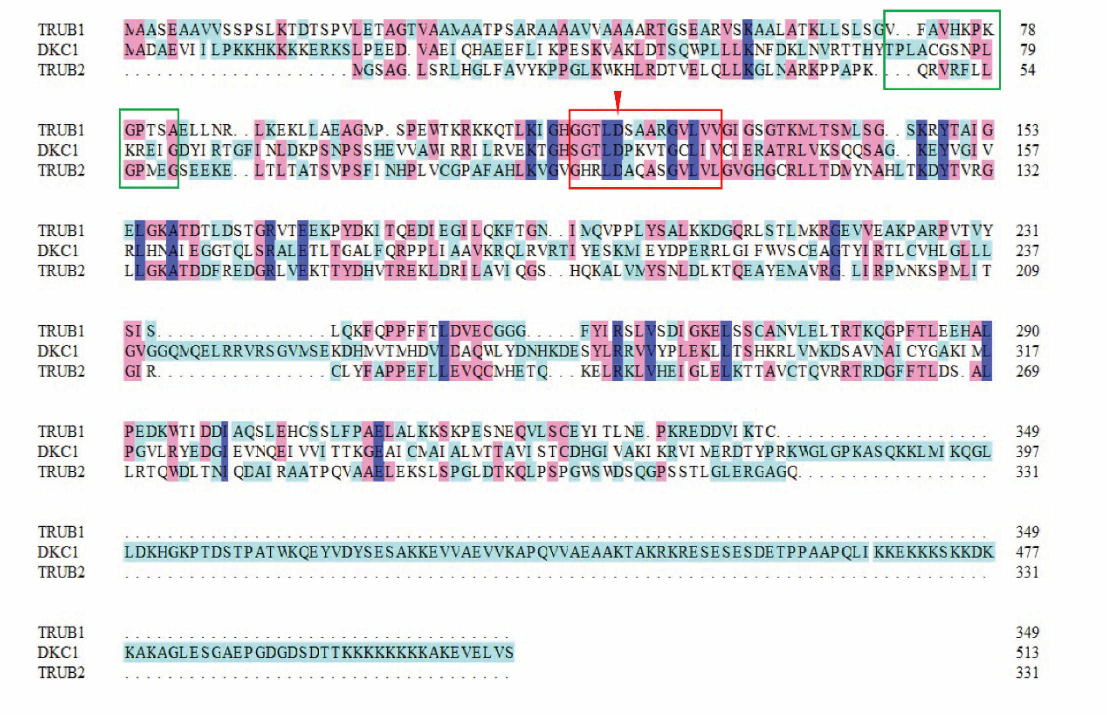

Figure S6. Sequence alignment of human TRUB1, DKC1 and TRUB2. The alignment was generated using the DNAMAN software. The corresponding accession numbers used for this analysis are as follows: TRUB1 (NP_631908.1), DKC1 (NP_001354.1) and TRUB2 (NP_056494.1). Numbers give the position of residues in proteins in relation to the first methionine of the TRUB1. Green box shows the motif I (VFAVHKPKGPTSA box in positions 71-83), the red box shows the motif II (GGTLDS AARGVLVV in positions 117-130) and the red triangle shows the highly conserved 121D residue of TRUB1. Amino acid residues shaded faint blue are identical; those shaded brick red are similar in at least two residues of 3 homologs, respectively.

Supplemental Table S1: DIG-labeled oligonucleotides used for primer extension assay

Name	Sequence (5'-3')	Description
ASN	DIG-TGGCTAGACCAATGG	Reverse transcription of m.tRNA^{Asn}
GLN	DIG-TGGCTAGGACTATGA	Reverse transcription of m.tRNA^{Gln}
GLU	DIG-TGGTATTCTCGCACG	Reverse transcription of m.tRNA^{Glu}
PRO	DIG-TGGTCAGAGAAAAAG	Reverse transcription of m.tRNA^{Pro}
MET	DIG-TGGTAGTACGGGAAG	Reverse transcription of m.tRNA^{Met}
LEUUUR	DIG-TGGTGTTAAGAAGAG	Reverse transcription of m.tRNA$^{Leu(UUR)}$
SERUCN	DIG-TGGCAAAAAAGGAAG	Reverse transcription of m.tRNA$^{Ser(UCN)}$
PHE	DIG-TGGTGTTTATGGGGT	Reverse transcription of m.tRNA^{Phe}
VAL	DIG-TGGTCAGAGCGGTCA	Reverse transcription of m.tRNA^{Val}
ILE	DIG-TGGTAGAAATAAGGG	Reverse transcription of m.tRNA^{Ile}
TRP	DIG-TGGCAGAAATTAAGT	Reverse transcription of m.tRNA^{Trp}
ALA	DIG-TGGTAAGGACTGCAA	Reverse transcription of m.tRNA^{Ala}
CYS	DIG-TGGAAGCCCCGGCAG	Reverse transcription of m.tRNA^{Cys}
TYR	DIG-TGGTGGTAAAAAGAG	Reverse transcription of m.tRNA^{Tyr}
ASP	DIG-TGGTAAGATATATAG	Reverse transcription of m.tRNA^{Asp}
LYS	DIG-TGGTCACTGTAAAGA	Reverse transcription of m.tRNA^{Lys}
GLY	DIG-TGGTACTCTTTTTTG	Reverse transcription of m.tRNA^{Gly}
ARG	DIG-TGGTTGGTAAATATG	Reverse transcription of m.tRNA^{Arg}
HIS	DIG-TGGGGTAAATAAGGG	Reverse transcription of m.tRNA^{His}
SERAGY	DIG-TGGTGAGAAAGCCAT	Reverse transcription of m.tRNA$^{Ser(AGY)}$
LEUCUN	DIG-TGGTACTTTTATTTG	Reverse transcription of m.tRNA$^{Leu(CUN)}$
THR	DIG-TGGTGTCCTTGGAAA	Reverse transcription of m.tRNA^{Thr}
HISCAC	DIG-TGGTGCCGTGACTCG	Reverse transcription of c.tRNA$^{His(CAC)}$
THRACU	DIG-TGGAGGCACCGCTGG	Reverse transcription of c.tRNA$^{Thr(ACU)}$
METAUG	DIG-TGGTGCCCTCTCTGA	Reverse transcription of c.tRNA$^{Met(AUG)}$
TYRUAC	DIG-TGGTCCTTCGAGCCG	Reverse transcription of c.tRNA$^{Tyr(UAC)}$

Supplemental Table 2. The bases (49-65) in the T-arms of mitochondrial tRNA^{Asn}, tRNA^{Gln}, tRNA^{Glu}, tRNA^{Pro}, tRNA^{Met}, tRNA$^{Leu(UUR)}$ and tRNA$^{Ser(UCN)}$.

Domains	T-stem					T-loop							T-stem				
Position	49	50	51	52	53	54	55	56	57	58	59	60	61	62	63	64	65
tRNA^{Asn}	G	U	G	G	G	m^{5}U	Ψ	U	A	A	G	U	C	C	C	A	U
tRNA^{Gln}	A	U	G	G	G	m^{5}U	Ψ	C	G	A	U	U	C	U	C	A	U
tRNA^{Glu}	m^{5}C	G	U	G	G	U	Ψ	G	U	A	G	U	C	C	G	U	G
tRNA^{Pro}	G	G	A	G		m^{5}U	Ψ	A	A	A	G	A		C	U	U	U
tRNA^{Met}	G	Ψ	U	G	G	U	Ψ	A	U	A	C		C	C	U	U	C
tRNA$^{Leu(UUR)}$	A	G	A	G	G	m^{5}U	Ψ	C	A	m^{1}A	U	U	C	C	U	C	U
tRNA$^{Ser(UCN)}$	G	G	G	G	G	m^{5}U	Ψ	C	G	m^{1}A	U	U	C	C	U	U	C

The gray background in the table shows the bases in the T-loop of tRNAs and the dark gray background indicates the U54, U55, and A58 in the T-loop of tRNAs.

Supplemental Table 3. Usage of asparagine, glutamic acid, glutamine and proline codons in human mitochondrial genes and average levels of individual polypeptide in *TRUB1* knockout cell lines, related to the average values in the control Hela cell line.

Proteins	Number of amino acids	Number of asparagine codons	Density of asparagine codons (%)	Number of glutamic acid codons	Density of glutamic acid codons (%)	Number of glutamine codons	Density of glutamine codons (%)	Number of proline codons	Density of proline codons (%)	Total number of 4 codons	Total density of 4 codons (%)	Relative levels of mitochondrial proteins (%)
ATP8	68	5	7.35	1	1.47	3	4.41	11	16.18	20	29.41	92.76
CO2	227	7	3.08	11	4.85	7	3.08	15	6.61	40	17.62	94.73
ND3	115	4	3.48	5	4.35	3	2.61	8	6.96	20	17.40	82.01
ND2	347	20	5.76	6	1.73	10	2.88	23	6.63	59	17.00	NA
ND1	318	13	4.09	11	3.46	6	1.89	22	6.92	52	16.36	73.10
ND5	603	33	5.47	9	1.49	20	3.32	32	5.31	94	15.59	80.00
ATP6	226	11	4.87	3	1.33	7	3.10	14	6.19	35	15.48	NA
ND4	459	23	5.01	9	1.96	10	2.18	23	5.01	65	14.16	70.17
CYTB	380	15	3.95	4	1.05	8	2.11	23	6.05	50	13.16	56.69
CO3	261	6	2.30	7	2.68	9	3.45	12	4.60	34	13.03	NA
CO1	513	17	3.31	10	1.95	6	1.17	29	5.65	62	12.08	NA
ND4L	98	6	6.12	2	2.04	1	1.02	2	2.04	11	11.22	104.37
ND6	174	4	2.30	10	5.75	0	0.00	5	2.87	19	10.92	NA

NA, not applicable.
